# Evaluation of whole-genome sequencing protocols for detection of antimicrobial resistance, virulence factors and mobile genetic elements in antimicrobial-resistant bacteria

**DOI:** 10.1099/jmm.0.001990

**Published:** 2025-03-19

**Authors:** Gabriel Cipriani, Karin Helmersen, Ricardo Ruiz Mazzon, Glauber Wagner, Hege Vangstein Aamot, Fabienne Antunes Ferreira

**Affiliations:** 1 Bacterial Molecular Genetics Laboratory (GeMBac), Department of Microbiology, Immunology, and Parasitology, Biological Sciences Center, Universidade Federal de Santa Catarina, Campus Universitário Reitor João David Ferreira Lima, Trindade, Postal Code 88040-960, Florianópolis, SC, Brazil; 2 Department of Microbiology and Infection Control, Akershus University Hospital, Lørenskog, Norway; 3 Department of Clinical Molecular Biology (EpiGen), Akershus University Hospital and University of Oslo, Lørenskog, Norway; 4 Bioinformatics Laboratory, Department of Microbiology, Immunology, and Parasitology, Biological Sciences Center, Campus Universitário Reitor João David Ferreira Lima, Trindade, Postal Code 88040-960, Florianópolis, SC, Brazil

**Keywords:** antibiotic resistance, bacteria, diagnosis, genome analysis, genotypic method, whole-genome sequencing

## Abstract

**Introduction.** Antimicrobial resistance (AMR) poses a critical threat to global health, underscoring the need for rapid and accurate diagnostic tools. Methicillin-resistant *Staphylococcus aureus* (MRSA) and extended-spectrum beta-lactamase (ESBL)-producing *Klebsiella pneumoniae* (ESBL-Kp) are listed among the World Health Organization’s priority pathogens.

**Hypothesis.** A rapid nanopore-based protocol can accurately and efficiently detect AMR genes, virulence factors (VFs) and mobile genetic elements (MGEs) in MRSA and ESBL-Kp, offering performance comparable to or superior to traditional sequencing methods.

**Aim.** Evaluate whole-genome sequencing (WGS) protocols for detecting AMR genes, VFs and MGEs in MRSA and ESBL-Kp, to identify the most accurate and efficient tool for pathogen profiling.

**Methodology.** Five distinct WGS protocols, including a rapid nanopore-based protocol (ONT20h) and four slower sequencing methods, were evaluated for their effectiveness in detecting genetic markers. The protocols' performances were compared across AMR genes, VFs and MGEs. Additionally, phenotypic antimicrobial susceptibility testing was performed to assess concordance with the genomic findings.

**Results.** Compared to four slower sequencing protocols, the rapid nanopore-based protocol (ONT20h) demonstrated comparable or superior performance in AMR gene detection and equivalent VF identification. Although MGE detection varied among protocols, ONT20h showed a high level of agreement with phenotypic antimicrobial susceptibility testing.

**Conclusion.** The findings highlight the potential of rapid WGS as a valuable tool for clinical microbiology, enabling timely implementation of infection control measures and informed therapeutic decisions. However, further studies are required to optimize the clinical application of this technology, considering costs, availability of bioinformatics tools and quality of reference databases.

**Impact Statement**

Rapid whole-genome sequencing using nanopore technology provides a promising solution for the timely detection of antimicrobial resistance (AMR) genes, virulence factors and mobile genetic elements in *Staphylococcus aureus* and *Klebsiella pneumoniae*. Our findings demonstrate that a rapid nanopore-based protocol delivers performance comparable to or superior to traditional sequencing methods, supporting its potential as an efficient diagnostic tool in clinical microbiology. This advancement could significantly improve infection control strategies and therapeutic decision-making, addressing the urgent need for faster, accurate pathogen profiling in the fight against AMR.

## Data Summary

The authors confirm that all supporting data, code and protocols have been provided within the article or through supplementary data files.

## Introduction

Antimicrobial resistance (AMR) represents a major global health challenge of the twenty-first century, as infections caused by antibiotic-resistant bacteria are associated with significant increases in morbidity and mortality rates, longer hospital stays and higher treatment costs [1]. Methicillin-resistant *Staphylococcus aureus* (MRSA) stands as one of the most common examples of AMR in Gram-positive bacteria, conferring resistance to nearly the entire class of beta-lactam antimicrobials [2,3]. MRSA can infect virtually any body site and is a leading cause of skin and soft tissue infections, bloodstream infections (BSIs) and osteoarticular infections [2,3]. Similarly, extended-spectrum beta-lactamase (ESBL)-producing *Klebsiella pneumoniae* (ESBL-Kp) is a prominent example of Gram-negative bacteria resistant to multiple antimicrobials, harbouring genes like *bla*_TEM_, *bla*_CTX-M_ and *bla*_OXA_, which confer resistance to monobactams and third- and fourth-generation cephalosporins. The widespread use of carbapenems to treat ESBL-Kp has led to the emergence of carbapenemase-producing strains, further complicating treatment [4,5]. *K. pneumoniae* is a major cause of nosocomial infections, including urinary tract infections, pneumonia and BSIs [5].

Despite ongoing efforts in developing new antibiotics, implementing active surveillance and advancing infection prevention strategies, those bacteria continue to pose a significant threat with enduringly high mortality rates [2,4]. The success of those pathogens is often attributed to the acquisition of mobile genetic elements (MGEs) associated with AMR, a diverse arsenal of virulence factors and other elements involved in niche adaptation [3,5]. Enhancing existing diagnostic methods is essential to curb the spread of antibiotic-resistant bacteria, such as MRSA and ESBL-Kp, in healthcare settings and to enable early and effective antimicrobial therapy for infected patients. Traditional culture-based techniques often cause delays in identifying AMR pathogens. Consequently, whole-genome sequencing (WGS) technology is being increasingly explored to improve clinical microbiology [6].

Over the past decade, advancements in sequencing technologies have made WGS increasingly accessible and rapid, driving the development of new methods for pathogen identification and characterization [7]. WGS has empowered clinical microbiologists with tools for detecting and monitoring AMR through WGS-AST (antimicrobial susceptibility testing). It also enables the detection of virulence factors and MGE such as plasmids, which are crucial for understanding the adaptability, pathogenicity and dissemination of AMR bacteria [6,7]. However, the widespread adoption of WGS in this context is hindered by several bioinformatics challenges, including sequencing quality control, accurate genome assembly and the availability of robust genomic analytical databases [6,7].

This study evaluates the effectiveness of different sequencing and assembly protocols in detecting AMR, virulence factors and MGEs in MRSA and ESBL-Kp. The evaluation includes a rapid WGS protocol based on Oxford Nanopore Technologies (ONT), developed by our research group for investigating AMR bacteria outbreaks [8]. The genomic analysis data generated are anticipated to advance the application potential of WGS protocols in clinical settings. The study hypothesizes that our previously developed genome sequencing and assembly protocol can detect genetic components as effectively as or better than traditional, slower sequencing methods.

## Methods

### Bacterial genomes

All bacterial isolates included in this study were sourced from the collection at the Department of Microbiology and Infection Control, Akershus University Hospital, University of Oslo, Norway. The draft genomes of 42 clinical MRSA isolates were included. These isolates were previously sequenced by our group [8] using both ONT and Illumina technology (IT) sequencing platforms. Additionally, six ESBL-Kp clinical isolates were included and subjected to sequencing using the protocols outlined in prior work [8].

This study analysed and compared five protocols (Table 1). The draft genomes were generated through three distinct sequencing protocols: two employed ONT (GridION) using the rapid barcoding kit (SQK-RBK004; ONT, Oxford, UK), with sequencing durations of 20 h (ONT20h) and 48 h (ONT48hA and ONT48hB) within the same run/flow cell (R9.4.1). One utilized IT performed on the Illumina MiSeq platform for 56 h (IT) using 2×300 bp paired-end sequencing. The ONT20h, ONT48B and IT protocols involved submitting raw data for assembly, following the methodology outlined by Ferreira *et al.* [8]. Briefly, the ONT20h and ONT48B protocols performed *de novo* assembly using Flye [9], followed by two rounds of polishing using Medaka (https://github.com/nanoporetech/medaka). The IT protocol performed *de novo* assembly on Geneious Prime software v2019.2.3 (https://www.geneious.com/) using BBDuk trimmer v1.0 and SPAdes v3.13.0. Additionally, two alternative protocols were employed: ONT48A, which underwent sequencing as previously described, but the assembly was conducted using Canu v.1.9 [10] without polishing, and Hybrid, which utilized raw data from both ONT20h and IT sequencing for assembly using Unicycler v.0.5.0 [11]. Hybrid genome assembly and ONT48A were exclusively performed for MRSA. Quality assessment of the assemblies was conducted using QUAST v.5 [12].

**Table 1.** Overview of sequencing and assembly protocols

Protocol	Technology/sequencing platform	Sequencing time (h)	*De novo* assembly software tool	Polishing software tool
ONT20h	ONT – GridION	20	Flye v.2.7.1	Medaka v.1.0.1
ONT48hA	ONT – GridION	48	Canu v.1.9	np
ONT48hB	ONT – GridION	48	Flye v.2.9	Medaka v.1.5.0
IT	IT – MiSeq	56	SPAdes v.3.13.0	np
Hybrid	ONT – GridION/IT – MiSeq	20/56*	Unicycler v.0.5.0	np

* Sequencing time for ONT and IT, respectively.

NPnot performed

### Genome analysis

AMR genes were identified using ResFinder [13] and CARD-RGI [14] for MRSA, and ResFinder for *K. pneumoniae*. Virulence-related genes, plasmids and other MGEs were detected using tools from the Center of Genomic Epidemiology (http://www.genomicepidemiology.org): VirulenceFinder [15], PlasmidFinder [16] and MobileElementFinder [17]. Since *K. pneumoniae* is absent from VirulenceFinder, the Virulence Factor Database (VFDB) [18] was used. All tools were run with default settings in their most recent versions for MRSA (Table S1, available in the online Supplementary Material) and *K. pneumoniae* (Table S2).

### Antimicrobial susceptibility testing

AST was conducted following the guidelines established by the European Committee on Antimicrobial Susceptibility Testing (EUCAST) [19]. MRSA isolates underwent testing against 16 antimicrobial agents using the disc diffusion method, including fusidic acid (10 µg), ampicillin (2 µg), penicillin G (1 µg), cefoxitin (30 µg), ciprofloxacin (5 µg), clindamycin (2 µg), chloramphenicol (30 µg), erythromycin (15 µg), gentamicin (10 µg), linezolid (10 µg), mupirocin (200 µg), oxacillin-1 (1 µg), rifampicin (5 µg), sulphamethoxazole-trimethoprim (25 µg), tetracycline (30 µg) and tigecycline (15 µg). Additionally, Etest^®^ was utilized to assess susceptibility to teicoplanin and vancomycin (AB Biodisk, Solna, Sweden). ESBL-Kp isolates were also evaluated using the disc diffusion method against 12 antimicrobial agents, including amoxicillin-clavulanate (20–10 µg), ampicillin (10 µg), aztreonam (30 µg), cefepime (30 µg), cefotaxime (5 µg), ceftazidime (10 µg), cefuroxime (30 µg), ciprofloxacin (5 µg), gentamicin (10 µg), meropenem (10 µg), piperacillin-tazobactam (30–6 µg), and sulphamethoxazole-trimethoprim (1.25–23.75 µg) (AB Biodisk, Solna, Sweden).

### Statistical analysis

Genomic analysis results were compared across protocols to evaluate their detection capabilities. Normality was assessed with the Shapiro–Wilk test, and non-parametric data were compared using the Kruskal–Wallis test, both in GraphPad Prism v.9.5.1 (https://www.graphpad.com/). Cohen’s kappa coefficient assessed agreement between genomic (resistance detection) and phenotypic AST results, calculated using VassarStats (http://vassarstats.net/).

Resistance inferred from genomic analyses was compared with phenotypic AST results to evaluate diagnostic accuracy. For this analysis, only genes with sequence lengths matching the reference at 97–100% identity were considered. Sensitivity, specificity, positive predictive value (PPV) and negative predictive value (NPV) were calculated to represent true positive and negative rates. A true positive was defined as concordant resistance by both methods, a false positive was resistance by genotype only, a false negative was resistance by phenotype only and a true negative was no resistance by either method. These analyses were conducted with MedCalc Software Ltd. Diagnostic test evaluation calculator v.22.009 (https://www.medcalc.org/calc/diagnostic_test.php).

## Results

### Overview of the sequenced data

In our study, we analysed 228 genomes from 48 clinical bacterial isolates. Ferreira *et al.* [8] sequenced and assembled all MRSA genomes from the ONT20h and IT protocols used in this study. They reported that DNA extraction and ONT library preparation took 5 h for six MRSA isolates per run, the same as for six *K. pneumoniae* isolates. For ONT-based sequencing, FASTQ files were obtained after 20 and 48 h. Assemblies from the 20 h protocol (ONT20h) averaged 40 min per MRSA isolate and 52 min per ESBL-Kp isolate. Assemblies for ONT48hA and ONT48hB took about 1 h and 10 min for both micro-organisms, whilst the hybrid protocol required an average of 4 h and 22 min for MRSA. Coverage for all genomes ranged from 71× to 946×. Detailed sequencing and assembly information is in the supplementary information (Tables S3 and S4).

### Genomic findings

#### Antimicrobial resistance

**Fig. 1.**
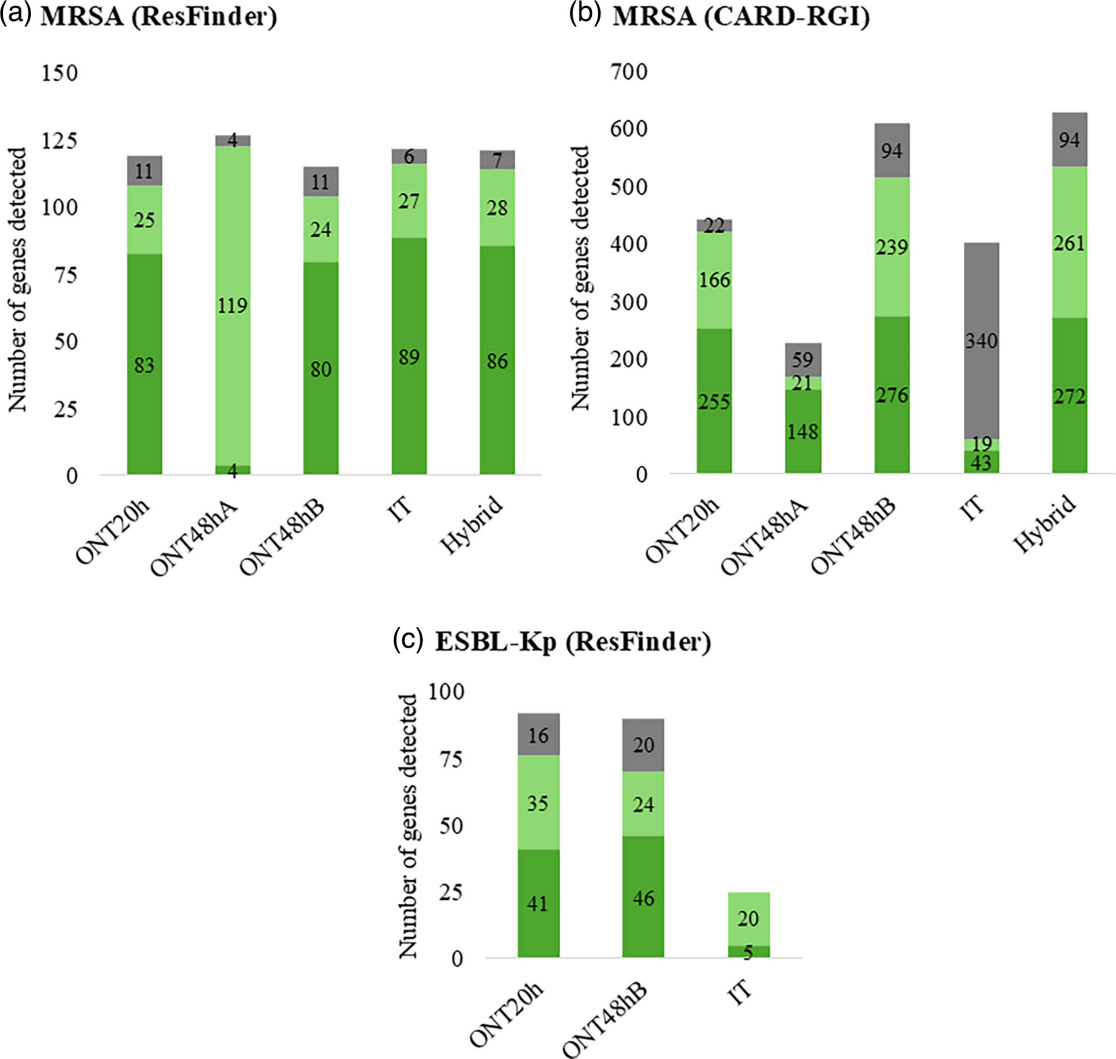
Charts showing the total number of AMR genes detected in MRSA and ESBL-Kp genomes. Genomes were obtained using five different protocols and analysed with ResFinder for MRSA (a), CARD-RGI for MRSA (b) and ResFinder for ESBL-Kp (c). Detected genes were classified into three categories: category A (dark green): sequence length in the genome matches the reference with 100% identity; category B (light green): sequence length in the genome matches the reference with <100% identity; category C (grey): sequence length in the genome does not match the reference, with ≤100% identity.

Analyses using ResFinder detected resistance genes in MRSA isolates for beta-lactams; tetracyclines; macrolides, lincosamides and streptogramins (MLS); aminoglycosides; and trimethoprim (Tables S5–S9). Gene detection varied qualitatively and quantitatively by genome, partly due to lineage. Overall, 127 antimicrobial genes were detected with ONT48hA, followed by IT (122), hybrid (121), ONT20h (119) and ONT48hB (115) (Fig. 1a), with no statistical difference in detection capacity between protocols (*P*=0.5211). CARD-RGI analyses showed significant protocol-dependent variations (Fig. 1b, Tables S10–S14). ONT48hB and hybrid had the highest detection rates, identifying 609 and 627 genes, respectively, compared to ONT20h (443 genes), IT (403 genes) and ONT48hA (228 genes). Statistical analysis showed differences in detection capacity (*P*<0.0001). ONT48hB and hybrid had high concordance with expected values (*P*>0.9999), whilst ONT20h, ONT48hA and IT deviated significantly (*P*<0.0001). For ESBL-Kp genomes, ResFinder identified genes related to resistance against various antibiotics, including aminoglycosides, phenicols, beta-lactams, disinfectants, fosfomycin, MLS, quinolones, sulphonamides, tetracyclines and trimethoprim (Tables S15–S17). The ONT20h and ONT48hB protocols detected similar numbers of resistance genes (90 and 92, respectively) (Fig. 1c). In contrast, the IT protocol detected significantly fewer genes (25) (*P*=0.0031). Statistical analysis confirmed that ONT20h and ONT48hB had high concordance with expected values (*P*>0.9999), whilst the IT protocol showed significant deviation (*P*=0.0031).

#### Virulence

**Fig. 2.**
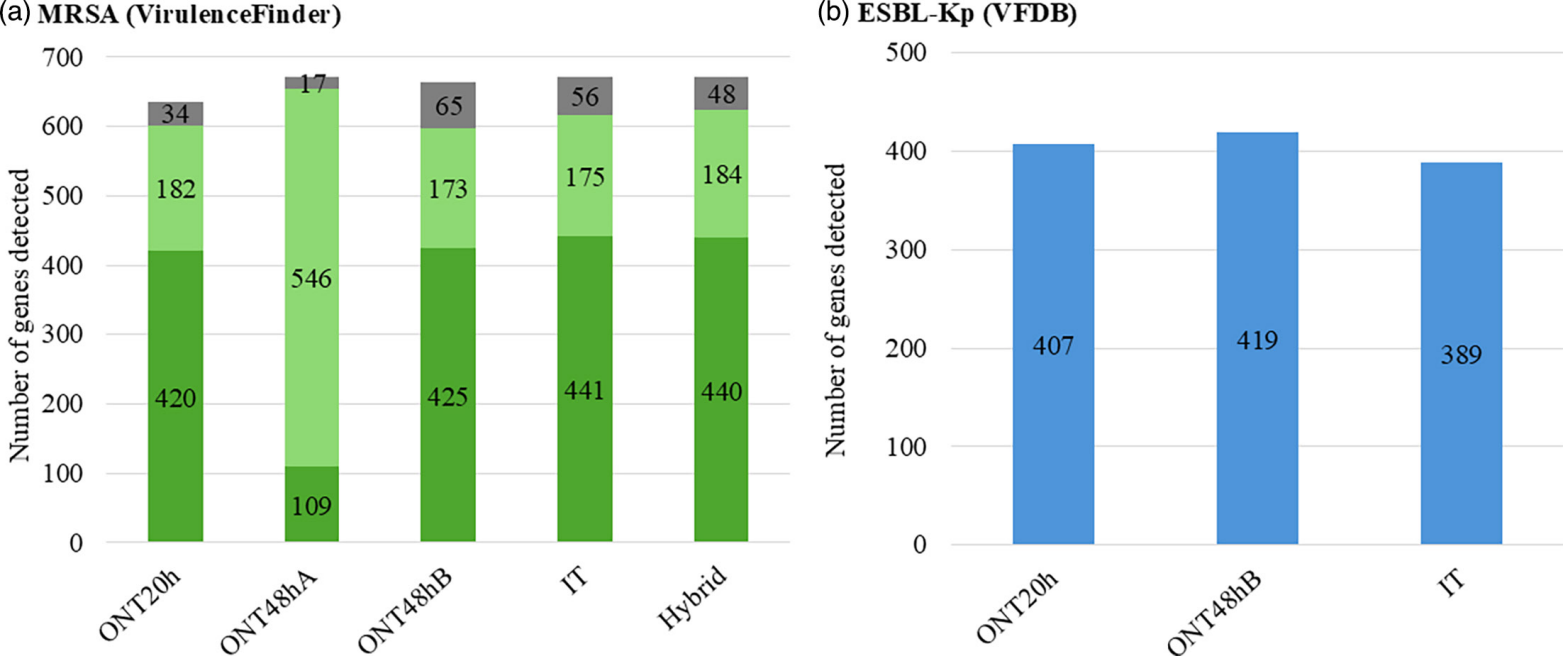
Chart showing the total number of virulence factor-related genes detected in MRSA genomes (a) and ESBL-Kp genomes (b). Analyses were performed using VirulenceFinder (a) and VFDB (b). Genes identified by VirulenceFinder were categorized as follows: category A (dark green): sequence length matches the reference with 100% identity; category B (light green): sequence length matches with <100% identity; category C (grey): sequence length does not match the reference, with ≤100% identity. VFDB results were categorized based on gene presence only.

Virulence factor analysis showed consistent detection across protocols (ONT48hA, IT and hybrid), with an average of 672 genes identified in MRSA genomes (Fig. 2a, Tables S18–S22). The ONT48hB protocol detected slightly fewer genes (663), followed by ONT20h (636 genes). No statistical difference in detection capacity was observed between protocols (*P*=0.9984). For ESBL-Kp genomes, VFDB analysis revealed a range of virulence factor genes. The ONT48hB protocol detected the most genes (419), followed by ONT20h (407) and IT (389) (Fig. 2b). There was no statistically significant difference in detection capacity among the protocols (*P*=0.1411).

#### Mobile genetic element

**Fig. 3.**
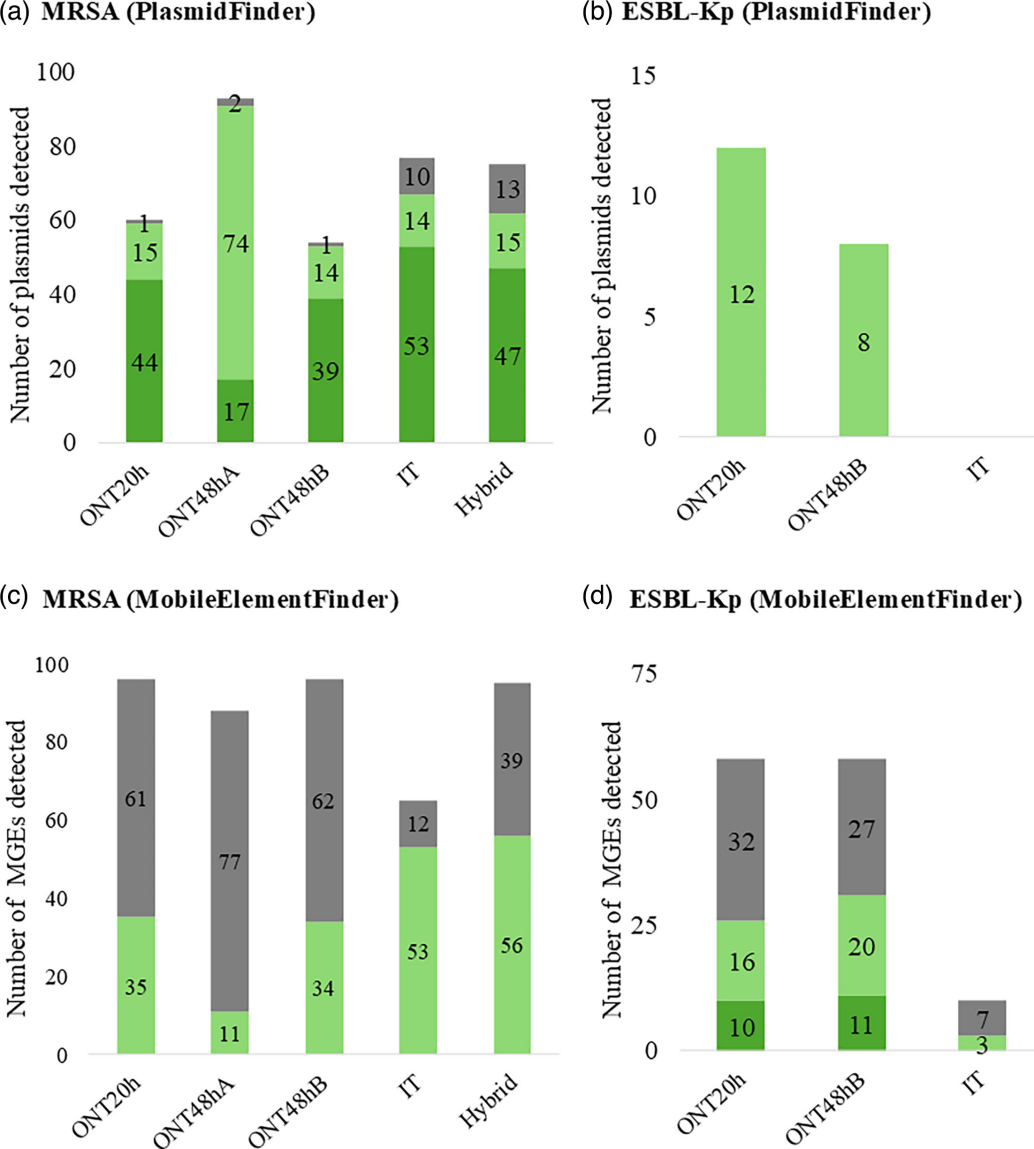
Charts showing the total number of plasmids (a and b) and MGEs, excluding plasmids (c and d), detected in MRSA genomes (a and c) and ESBL-Kp genomes (b and d). Analyses were performed using PlasmidFinder for plasmids and MobileElementFinder for MGEs. Elements identified were categorized as follows: category A (dark green): sequence length in the genome matches the reference with 100% identity; category B (light green): sequence length in the genome matches the reference with <100% identity; category C (grey): sequence length in the genome does not match the reference, with ≤100% identity.

PlasmidFinder identified a variable number of plasmids in MRSA genomes across protocols (Fig. 3a, Tables S23–S27). The ONT48hA protocol captured the highest number (93 plasmids), followed by IT (77), hybrid (74), ONT20h (59) and ONT48hB (54). Statistical analysis revealed a significant difference in plasmid detection capacity between protocols (*P*=0.0005). Interestingly, only the ONT20h and ONT48hB protocols deviated significantly from the expected results (*P*=0.007 and *P*=0.0015, respectively). Analysis using PlasmidFinder revealed a striking disparity in plasmid detection between protocols for ESBL-Kp genomes (Fig. 3b, Tables S28–S30). Notably, all ONT-based protocols identified plasmids, whereas the IT protocol yielded no detections. This significant difference in detection capacity was statistically confirmed (*P*=0.0002). Furthermore, the IT protocol was the only one deviating significantly from the expected results (*P*=0.0003).

MobileElementFinder analysis revealed the presence of insertion sequences across all MRSA and ESBL-Kp genomes (Tables S23–S30). Both datasets showed a similar trend: the ONT20h and ONT48hB protocols consistently detected the highest number of MGEs (MRSA: 96; ESBL-Kp: 58) (Fig. 3c, d). This was followed by the hybrid (MRSA: 95), ONT48hA (MRSA: 88) and IT protocols (MRSA: 65; ESBL-Kp: 10). Statistical analysis confirmed significant differences in detection capacity between protocols for both MRSA (*P*=0.0466) and ESBL-Kp (*P*=0.0026) genomes. The IT protocol showed lower performance and significant deviation from the expected results in both analyses (MRSA: *P*=0.0326; ESBL-Kp: *P*=0.0014).

When analysing the combined results from PlasmidFinder and MobileElementFinder, a significant difference in detection capacity between protocols was observed for both MRSA (*P*=0.0496) and ESBL-Kp (*P*=0.0019) genomes. Notably, the IT protocol yielded significantly fewer elements compared to expected values in both analyses (MRSA: *P*=0.0297; ESBL-Kp: *P*=0.0008). Conversely, the other protocols displayed concordance with the expected results.

#### Phenotypic and WGS-AST

Phenotypic AST was performed on 36 of the 42 MRSA isolates. As expected, all isolates exhibited resistance to beta-lactam antibiotics. Tetracycline resistance was detected in 12 isolates (33.3%). Four isolates (11.1%) showed gentamicin resistance, with two (5.6%) also resistant to erythromycin. Ciprofloxacin resistance was identified in two isolates (5.6%), whilst the remaining were sensitive. Fusidic acid resistance was found in another two isolates (5.6%). All isolates were susceptible to the other antimicrobials tested (Table S31). All ESBL-Kp isolates exhibited broad phenotypic resistance to amoxicillin-clavulanate, ampicillin, aztreonam, cefepime, cefotaxime, ceftazidime, cefuroxime and sulphamethoxazole-trimethoprim. Notably, 66.7% of the isolates (four out of six) also showed resistance to ciprofloxacin, whilst the remaining 33.3% (two out of six) were susceptible under increased exposure. One isolate (16.7%) exhibited susceptibility to piperacillin-tazobactam with increased exposure. All isolates remained susceptible to gentamicin and meropenem (Table S32). Table 2 presents detailed quantitative susceptibility data from both phenotypic and WGS-AST analyses. This table provides a comparative overview of resistance patterns, allowing for an evaluation of both phenotypic and genotypic resistance across different bacterial species and testing protocols.

**Table 2.** Comparison of phenotypic and genotypic AST results presented for MRSA and ESBL-Kp genomes across different protocols

MRSA				
(a) Protocols ONT20h, IT and hybrid		WGS-AST		
		Resistant	Susceptible	Total
Phenotypic AST	Resistant	170 (TP)	0 (FN)	170
	Susceptible	0 (FP)	478 (TN)	478
	Total	170	478	648
(b) Protocol ONT48A		WGS-AST		
		Resistant	Susceptible	Total
Phenotypic AST	Resistant	168 (TP)	2 (FN)	170
	Susceptible	5 (FP)	473 (TN)	478
	Total	173	475	648
(c) Protocol ONT48B		WGS-AST		
		Resistant	Susceptible	Total
Phenotypic AST	Resistant	169 (TP)	1 (FN)	170
	Susceptible	0 (FP)	478 (TN)	478
	Total	169	479	648
**ESBL-Kp**				
(a) Protocols ONT20h and ONT48B		WGS-AST		
		Resistant	Susceptible	Total
Phenotypic AST	Resistant	52 (TP)	0 (FN)	52
	Susceptible	5 (FP)	15 (TN)	20
	Total	57	15	72
(b) Protocol IT		WGS-AST		
		Resistant	Susceptible	Total
Phenotypic AST	Resistant	46 (TP)	6 (FN)	52
	Susceptible	2 (FP)	18 (TN)	20
	Total	48	24	72

FN, false negatives; FP, false positives; TN, true negatives; TP, true positives

WGS-AST demonstrated high accuracy in predicting resistance profiles for MRSA. The ONT20h, IT and hybrid protocols achieved perfect concordance with phenotypic testing, showing 100% sensitivity, specificity, PPV and NPV (Table 3). However, the ONT48hA and ONT48hB protocols showed decreased performance due to false negatives for tetracycline and false positives for tetracycline and fusidic acid, leading to lower accuracy (Table 2). Overall, WGS-AST accuracy exceeded 98%, with ONT20h, IT and hybrid protocols achieving 100% accuracy. Cohen’s kappa was 1 for ONT20h, IT and hybrid, and 0.97 and 0.99 for ONT48hA and ONT48hB, respectively. For ESBL-Kp, ONT20h and ONT48hB protocols demonstrated the highest sensitivity (100%) and NPV (Table 3), effectively detecting all true isolates. However, these protocols had reduced specificity (75%) due to five false positives for ciprofloxacin and piperacillin-tazobactam. The IT protocol showed lower sensitivity (88.46%) with six false negatives for sulphamethoxazole-trimethoprim and two false positives for ciprofloxacin (Table 3). Despite maintaining high specificity (90%) and PPV (95.83%), the IT protocol had lower concordance (Cohen’s kappa: 0.73) compared to ONT20h and ONT48hB (0.81) (Table 3).

**Table 3.** Accuracy metrics of AST based on genotypic methods on MRSA and ESBL-Kp genomes

MRSA			
Parameters	ONT20h/IT/hybrid	ONT48hA	ONT48hB
Sensibility (CI 95%)	100% (97.85–100)	98.82% (95.81–99.86)	99.41% (96.77–99.99)
Specificity (CI 95%)	100% (99.23–100)	98.95% (97.58–99.66)	100% (99.23–100)
PPV (CI 95%)	100% (97.85–100)	97.11% (93.35–98.77)	100% (97.84–100)
NPV (CI 95%)	100% (99.23–100)	99.58% (98.35–99.89)	99.79% (98.54–99.97)
Accuracy	100% (99.43–100)	98.92% (97.79–99.56)	99.85% (99.14–100)
Cohen’s kappa (CI 95%)	1 (1–1)	0.97 (0.95–0.99)	0.99 (0.98–1)
**ESBL-Kp**			
Parameters	ONT20h/ONT48B	IT	
Sensibility (CI 95%)	100% (93.15–100)	88.46% (76.56–95.65)	
Specificity (CI 95%)	75% (50.90–91.34)	90% (68.30–98.77)	
PPV (CI 95%)	91.23% (82.96–95.69)	95.83% (86.02–98.85)	
NPV (CI 95%)	100% (78.20–100)	75% (58.22–86.59)	
Accuracy	93.06% (84.73–97.71)	88.89% (79.28–95.08)	
Cohen’s kappa (CI 95%)	0.81 (0.65–0.96)	0.73 (0.57–0.90)	

## Discussion

The performance of sequencing/assembly protocols for detecting AMR and virulence factor genes varied. We considered that a higher percentage of category A (green) indicates better protocol performance. Notably, the ONT20h protocol achieved comparable or superior accuracy compared to slower protocols in detecting AMR and virulence-related genes. Furthermore, ONT20h proved effective for identifying MGE. However, plasmid detection was lower with ONT20h compared to other protocols, surpassing only ONT48hB.

Safar *et al.* [20] examined the impact of *de novo* assembly and polishing tools on ONT-based bacterial genomes, showing how tool choice affects genome size, AMR/virulence gene detection and plasmid identification. Flye assemblies with polishing excelled in AMR/virulence gene detection, whilst Canu performed better for plasmid identification. Consistent with these findings, our study also emphasized the importance of tool selection. For example, the ONT48hA protocol yielded more category B (sequence length in the genome matches the reference with <100% identity) results than others, despite using the same reads as ONT20h, suggesting that assembly/polishing processes affect final genome quality. Canu, used in ONT48hA, may be less efficient in default mode, but optimized parameters improve metrics like N50 and reduce contigs and gaps [21]. As in other studies [21,22], we highlight the role of polishing tools like Medaka in enhancing genome quality and detecting genetic elements.

Whilst circular bacterial chromosomes are easier to assemble with long reads from ONT or PacBio, Illumina short reads can struggle with repetitive regions in MGEs and high G+C content [23]. Long reads offer better contiguity but are prone to errors and may miss small plasmids. Hybrid approaches, combining long and short reads, have shown better contiguity, longer contigs and improved accuracy compared to using only short or long reads [23,24]. In this study, the hybrid protocol consistently produced results statistically similar to other protocols and expected outcomes, highlighting its accuracy. However, this approach may not be ideal for urgent diagnostics due to its longer time requirements.

WGS-AST using the ONT20h protocol achieved 100% accuracy for MRSA, demonstrating its effectiveness. For ESBL-Kp, accuracy was 93.06%, with 100% sensitivity but lower specificity (75%) due to false positives. This highlights a key limitation of genotypic AST: the presence of resistance genes does not always correlate with phenotypic resistance, as unexpressed genes may not increase the MIC, leading to misclassification [23]. Our findings align with studies reporting high concordance between genotypic and phenotypic AST for certain antimicrobials [25,26], though this concordance can vary significantly (up to 20% or more) depending on the agent [6,27]. This variation illustrates the complex genotype–phenotype relationship, which remains challenging for accurate resistance prediction. Whilst some resistance mechanisms are well understood, others require further research to improve prediction accuracy. This limitation can reduce target detection and increase the risk of false negatives [27], emphasizing the need for foundational research to refine resistance prediction from WGS data [28]. In 2017, a EUCAST subcommittee critically evaluated WGS for AST, identifying a major limitation: the lack of robust evidence for its clinical effectiveness [26]. They called for further research and international collaboration to develop standardized bioinformatics tools, quality control measures and performance standards. The report also stressed the need for a continuously updated, unified database of AMR-associated genes and mutations. Since then, significant progress has been made in these areas.

An interlaboratory study assessed the reproducibility of genotypic AMR predictions and identified sources of discordance [29]. Key factors included sequencing data quality, bioinformatics methods and result interpretation. Low-quality sequencing and insufficient coverage adversely affected AMR gene identification, reducing sensitivity and specificity. Additionally, variations in analytical tools, parameters and reference databases led to inconsistencies in genetic variant identification. Doyle *et al.* [29] emphasized the need for high identity parameters and coverage thresholds to minimize errors. These findings highlight concerns about the reliability of current bioinformatics tools for clinical AMR prediction and underscore the need for standardized, user-friendly analytical tools and databases for consistent clinical applications [28]. An ISO-certified bioinformatics platform, abriTAMR, has been recently developed to detect AMR genes from WGS data. This platform leverages the National Center for Biotechnology Information AMRFinderPlus database and presents its results in a user-friendly report format, enhancing their utility and comprehension in clinical settings. During validation, abriTAMR demonstrated exceptional performance, achieving 99.9% accuracy, with high sensitivity (98.9%; 95% CI, 98.4–99.3) and high specificity (98.9%; 95% CI, 98.7–99.1) [30].

Our study demonstrated the accuracy and concordance of WGS-AST with phenotypic data using the rapid ONT20h protocol, underscoring its potential for clinical application due to its significantly faster turnaround time – a critical advantage for managing infections with suspected resistance. Despite these promising results, the study’s small sample size, particularly for ESBL-Kp, is a limitation. Future research should involve larger, more diverse sample sets, including a broader range of bacterial species, geographical locations and AMR profiles. For the clinical adoption of genomic protocols, further investigations are needed to address challenges such as cost, standardization, streamlined workflows and the accuracy of bioinformatics tools and databases.

## supplementary material

10.1099/jmm.0.001990 Uncited Supplementary Material 1.

## Abbreviations

AMR: antimicrobial resistance
BSIs: bloodstream infections
ESBL: extended-spectrum beta-lactamase
ESBL-Kp: extended-spectrum beta-lactamase-producing *Klebsiella pneumoniae*
EUCAST: European Committee on Antimicrobial Susceptibility Testing
IT: Illumina technology
MGEs: mobile genetic elements
MLS: macrolides, lincosamides and streptogramins
MRSA: methicillin-resistant *Staphylococcus aureus*
NPV: negative predictive value
ONT: Oxford Nanopore Technologies
PPV: positive predictive value
VFDB: Virulence Factor Database
VFs: virulence factors
WGS: whole-genome sequencing
