# Supplementary material for: Evaluation of whole-genome sequencing protocols for detection of antimicrobial resistance, virulence factors and mobile genetic elements in antimicrobial-resistant bacteria
Source: J Med Microbiol. 2025 Mar 19;74(3):001990. doi: 10.1099/jmm.0.001990 (PMC11923095; doi:10.1099/jmm.0.001990)
Supplement: Uncited Supplementary Material 1. [file jmm-74-01990-s001.pdf]

Cipriani et al. Evaluation of whole genome sequencing protocols for detection of antimicrobial resistance, virulence factors, and mobile genetic elements in antimicrobial-resistant bacteria

Supplementary material

Summary

Table S1. Versions of the services and their respective databases used for genomic analyses of MRSA genomes, in accordance with sequencing protocols. 3

Table S2. Versions of the services and their respective databases used for genomic analyses of ESBL-Kp genomes, in accordance with sequencing protocols. 4

Table S3. Information on Genome Sequencing. Average read length, number of reads analyzed, number of bases sequenced (Gb), and average Quality Score (Q-score) for sequencing protocols performed on each bacterial isolate using Oxford Nanopore Technology (ONT) with 20 and 48 hours of sequencing and Illumina Technology (IT). 5

Table S4. Information on Genome Assembly. Assembled genome size, number of contigs, largest contig size, GC content percentage (%), N50, L50, and coverage for the genomes of each bacterial isolate obtained using different sequencing/assembly protocols. 9

Table S5. Table of results from genomic analyses conducted using the ResFinder service with MRSA genomes obtained through the ONT20h protocol. 14

Table S6. Table of results from genomic analyses conducted using the ResFinder service with MRSA genomes obtained through the ONT48hA protocol. 15

Table S7. Table of results from genomic analyses conducted using the ResFinder service with MRSA genomes obtained through the ONT48hB protocol. 16

Table S8. Table of results from genomic analyses conducted using the ResFinder service with MRSA genomes obtained through the IT protocol. 17

Table S9. Table of results from genomic analyses conducted using the ResFinder service with MRSA genomes obtained through the Hybrid protocol. 18

Table S10. Table of results from genomic analyses conducted using the CARD-RGI service with MRSA genomes obtained through the ONT20h protocol. 19

Table S11. Table of results from genomic analyses conducted using the CARD-RGI service with MRSA genomes obtained through the ONT48hA protocol. 20

Table S12. Table of results from genomic analyses conducted using the CARD-RGI service with MRSA genomes obtained through the ONT48hB protocol. 21

Table S13. Table of results from genomic analyses conducted using the CARD-RGI service with MRSA genomes obtained through the IT protocol. 22

Table S14. Table of results from genomic analyses conducted using the CARD-RGI service with MRSA genomes obtained through the Hybrid protocol. 23

Table S15.Table of results from genomic analyses conducted using the ResFinder service with ESBL-Kp genomes obtained through the ONT20h protocol. 24

Table S16.Table of results from genomic analyses conducted using the ResFinder service with ESBL-Kp genomes obtained through the ONT48hB protocol. 24

Table S17.Table of results from genomic analyses conducted using the ResFinder service with ESBL-Kp genomes obtained through the IT protocol. 24

Table S18. Table of results from genomic analyses conducted using the VirulenceFinder service with MRSA genomes obtained through the ONT20h protocol. 25
Table S19. Table of results from genomic analyses conducted using the VirulenceFinder service with MRSA genomes obtained through the ONT48hA protocol. 26
Table S20. Table of results from genomic analyses conducted using the VirulenceFinder service with MRSA genomes obtained through the ONT48hB protocol. 27
Table S21. Table of results from genomic analyses conducted using the VirulenceFinder service with MRSA genomes obtained through the IT protocol. 28
Table S22. Table of results from genomic analyses conducted using the VirulenceFinder service with MRSA genomes obtained through the Hybrid protocol. 29
Table S23. Table of results from genomic analyses conducted using the PlasmidFinder and MobileElementFinder services with MRSA genomes obtained through the ONT20h protocol. 30
Table S24. Table of results from genomic analyses conducted using the PlasmidFinder and MobileElementFinder services with MRSA genomes obtained through the ONT48hA protocol. 31
Table S25. Table of results from genomic analyses conducted using the PlasmidFinder and MobileElementFinder services with MRSA genomes obtained through the ONT48hB protocol. 32
Table S26. Table of results from genomic analyses conducted using the PlasmidFinder and MobileElementFinder services with MRSA genomes obtained through the IT protocol. 33
Table S27. Table of results from genomic analyses conducted using the PlasmidFinder and MobileElementFinder services with MRSA genomes obtained through the Hybrid protocol. 34
Table S28. Table of results from genomic analyses conducted using the PlasmidFinder and MobileElementFinder services with ESBL-Kp genomes obtained through the ONT20h protocol. 35
Table S29. Table of results from genomic analyses conducted using the PlasmidFinder and MobileElementFinder services with ESBL-Kp genomes obtained through the ONT48hB protocol. 35
Table S30. Table of results from genomic analyses conducted using the PlasmidFinder and MobileElementFinder services with ESBL-Kp genomes obtained through the IT protocol. 36
Table S31. Results for Phenotypic Antimicrobial Susceptibility Testing Conducted on Clinical Isolates of MRSA. S = Susceptible (standard dosing); I = Susceptible (increased exposure); R = Resistant. Values in parentheses represent inhibition zone diameters in millimeters. 37
Table S32. Results for Phenotypic Antimicrobial Susceptibility Testing Conducted on Clinical Isolates of ESBL-Kp. S = Susceptible (standard dosing); I = Susceptible (increased exposure); R = Resistant. Values in parentheses represent inhibition zone diameters in millimeters. 38

Table S1. Versions of the services and their respective databases used for genomic analyses of MRSA genomes, in accordance with sequencing protocols.

Sequencing Protocol	Service	Version of the service	Version of the database
ONT20h	*ResFinder*	2021-05-27	2021-04-20
	VirulenceFinder	2.0.3 (2020-05-21)	2020-05-29
	PlasmidFinder	2.0.1 (2020-07-01)	2021-07-12
	MobileElementFinder	1.0.3 (2020-10-09)	1.0.2 (2020-06-09)
	CARD-RGI	5.2.0	3.1.4
ONT48hA	*ResFinder*	2021-05-27	2021-09-22
	VirulenceFinder	2.0.3 (2020-05-21)	2020-05-29
	PlasmidFinder	2.0.1 (2020-07-01)	2021-07-12
	MobileElementFinder	1.0.3 (2020-10-09)	1.0.2 (2020-06-09)
	CARD-RGI	5.2.0	3.1.4
ONT48hB	*ResFinder*	2022-03-10	2022-02-04
	VirulenceFinder	2.0.2 (2020-05-21)	2020-05-29
	PlasmidFinder	2.0.1 (2020-07-01)	2021-11-29
	MobileElementFinder	1.0.3 (2020-10-09)	1.0.2 (2020-06-09)
	CARD-RGI	6.0.0	3.2.5
IT	*ResFinder*	2021-05-27	2021-04-20
	VirulenceFinder	2.0.3 (2020-05-21)	2020-05-29
	PlasmidFinder	2.0.1 (2020-07-01)	2021-07-12
	MobileElementFinder	1.0.3 (2020-10-09)	1.0.2 (2020-06-09)
	CARD-RGI	5.2.0	3.1.4
Hybrid	*ResFinder*	2022-08-08	2022-05-24
	VirulenceFinder	2.0.3 (2020-05-21)	2022-12-02
	PlasmidFinder	2.0.1 (2020-07-01)	2023-01-18
	MobileElementFinder	1.0.3 (2020-10-09)	1.0.2 (2020-06-09)
	CARD-RGI	6.0.0	3.2.5

Table S2. Versions of the services and their respective databases used for genomic analyses of ESBL-Kp genomes, in accordance with sequencing protocols.

**Sequencing Protocol**	**Service**	**Version of the service**	**Version of the database**
ONT20h	*ResFinder*	2022-08-08	2022-05-24
	VFDB	5 (2023-04-05)	5 (2023-04-05)
	PlasmidFinder	2.0.1 (2020-07-01)	2023-01-18
	MobileElementFinder	1.0.3 (2020-10-09)	1.0.2 (2020-06-09)
ONT48hB	*ResFinder*	2022-08-08	2022-05-24
	VFDB	5 (2023-06-28)	5 (2023-06-28)
	PlasmidFinder	2.0.1 (2020-07-01)	2023-01-18
	MobileElementFinder	1.0.3 (2020-10-09)	1.0.2 (2020-06-09)
IT	*ResFinder*	2022-08-08	2022-05-24
	VFDB	5 (2023-04-05)	5 (2023-04-05)
	PlasmidFinder	2.0.1 (2020-07-01)	2023-01-18
	MobileElementFinder	1.0.3 (2020-10-09)	1.0.2 (2020-06-09)

Table S3. Information on Genome Sequencing. Average read length, number of reads analyzed, number of bases sequenced (Gb), and average Quality Score (Q-score) for sequencing protocols performed on each bacterial isolate using Oxford Nanopore Technology (ONT) with 20 and 48 hours of sequencing and Illumina Technology (IT).

**Isolate**	**Sequencing protocol**	**Read length (mean; bp)**	**N° reads analysed**	**Yield (gb)**	**Q-score (mean)**
MRSA_01	ONT 20h	3869	375063	1.4	13.1
	ONT 48h	3858	573267	2.21	11.6
	IT	152	1754542		
MRSA_02	ONT 20h	1328	343980	0.456	11.9
	ONT 48h	1263	646435	0.817	10.5
	IT	157	939052		
MRSA_03	ONT 20h	2374	281897	0.669	12.3
	ONT 48h	2286	394129	0.901	11.2
	IT	161	1140438		
MRSA_04	ONT 20h	1962	202473	0.397	13.6
	ONT 48h	1956	308839	0.604	12
	IT	155	1042266		
MRSA_05	ONT 20h	1524	271438	0.414	11.9
	ONT 48h	1451	495348	0.718	10.7
	IT	240	1397759		
MRSA_06	ONT 20h	2222	355173	0.789	11.7
	ONT 48h	2101	689164	1.44	10.3
	IT	219	1653672		
MRSA_07	ONT 20h	2393	387687	0.928	12.32
	ONT 48h	2314	536596	1.24	11.2
	IT	200	1711446		
MRSA_08	ONT 20h	3715	259388	0.963	11.7
	ONT 48h	3545	497725	1.76	10.5
	IT	158	1568694		
MRSA_09	ONT 20h	4061	558430	2.2	11.8
	ONT 48h	3886	1095744	4.25	10.5
	IT	200	1454580		
MRSA_10	ONT 20h	3383	310661	1	12.7
	ONT 48h	3383	470819	1.59	10.9
	IT	128	1326662		
MRSA_11	ONT 20h	3181	110745	0.352	11.7
	ONT 48h	3098	216997	0.672	10.5
	IT	209	1779820		
MRSA_12	ONT 20h	2520	187923	0.473	12.9
	ONT 48h	2523	274607	0.692	11.2
	IT	129	2196378		
MRSA_13	ONT 20h	5959	185714	1.1	13.4
	ONT 48h	6008	217441	1.3	12
	IT	209	1628272		

MRSA_14	ONT 20h	2764	149504	0.413	13.3
	ONT 48h	2763	174381	0.487	11.8
	IT	218	1382044		
MRSA_15	ONT 20h	1880	189497	0.356	12.35
	ONT 48h	1779	265153	0.471	11.2
	IT	206	1505182		
MRSA_16	ONT 20h	6097	459081	2.7	13.5
	ONT 48h	6048	883811	5.34	11.7
	IT	202	1822354		
MRSA_17	ONT 20h	4777	319204	1.5	12.8
	ONT 48h	4066	472850	1.92	11.2
	IT	190	1206720		
MRSA_18	ONT 20h	4172	337536	1.4	12.4
	ONT 48h	4755	489413	2.32	11.1
	IT	213	1364524		
MRSA_19	ONT 20h	3467	289439	1	13.6
	ONT 48h	3521	546204	1.92	11.9
	IT	212	1736968		
MRSA_20	ONT 20h	3833	203478	0.78	12.7
	ONT 48h	3826	307739	1.17	10.9
	IT	205	1116512		
MRSA_21	ONT 20h	3458	362110	1.2	12.8
	ONT 48h	3454	540867	1.86	11
	IT	201	1715146		
MRSA_22	ONT 20h	5040	150883	0.76	13.4
	ONT 48h	5051	176225	0.89	11.9
	IT	231	1681230		
MRSA_23	ONT 20h	3674	169465	0.622	13.3
	ONT 48h	3690	199735	0.737	11.7
	IT	221	1463374		
MRSA_24	ONT 20h	4665	152432	0.711	13.4
	ONT 48h	4697	214092	1	11.9
	IT	229	1671346		
MRSA_25	ONT 20h	5234	149649	0.783	13.4
	ONT 48h	5250	205691	1.07	11.9
	IT	232	1309892		
MRSA_26	ONT 20h	5944	250146	1.4	13.2
	ONT 48h	5996	353984	2.12	11.7
	IT	222	1585422		
MRSA_27	ONT 20h	5403	228273	1.2	13.3
	ONT 48h	5424	314485	1.7	11.8
	IT	244	1119728		
MRSA_28	ONT 20h	6008	222342	1.3	13.4
	ONT 48h	6013	309808	1.86	11.9
	IT	255	1269168		
MRSA_29	ONT 20h	3772	448882	1.7	13.3
	ONT 48h	3771	718085	2.7	11.7
	IT	233	1238678		
	ONT 20h	4618	294540	1.3	13.5

MRSA_30	ONT 48h	4620	463757	2.14	12
	IT	219	1415030		
MRSA_31	ONT 20h	6370	278437	1.7	13.5
	ONT 48h	6367	445495	2.83	11.8
	IT	210	1238686		
MRSA_32	ONT 20h	2170	232323	0.504	13.6
	ONT 48h	2162	356195	0.77	11.9
	IT	204	1933856		
MRSA_33	ONT 20h	3940	90521	0.356	13.3
	ONT 48h	3950	105003	0.414	11.8
	IT	230	1709504		
MRSA_34	ONT 20h	8468	158779	1.3	13.4
	ONT 48h	8421	250910	2.11	11.7
	IT	194	2120956		
MRSA_35	ONT 20h	4445	264142	1.1	13.5
	ONT 48h	4448	499991	2.22	11.7
	IT	218	1669562		
MRSA_36	ONT 20h	4318	285407	1.2	13.5
	ONT 48h	4304	544303	2.34	11.6
	IT	227	1393224		
MRSA_37	ONT 20h	5303	194251	1	13.6
	ONT 48h	5351	360746	1.93	11.9
	IT	220	1031474		
MRSA_38	ONT 20h	4041	262256	1	13.4
	ONT 48h	4017	493300	1.98	11.5
	IT	220	1058394		
MRSA_39	ONT 20h	2544	318509	0.81	12.35
	ONT 48h	2452	450253	1.1	11.1
	IT	238	1365038		
MRSA_40	ONT 20h	3551	434669	1.5	13.1
	ONT 48h	3546	665541	2.36	11.6
	IT	230	1136686		
MRSA_41	ONT 20h	3575	384704	1.3	13.2
	ONT 48h	3582	587427	2.1	11.7
	IT	213	1164138		
MRSA_42	ONT 20h	3044	137974	0.42	13.4
	ONT 48h	3065	183909	0.563	11.8
	IT	236	1713648		
Kp_01	ONT 20h	6328	64000	0.405	11.8
	ONT 48h	6140	91607	0.562	10.5
	IT	231.9	2031126		
Kp_02	ONT 20h	7630	127381	0.972	12.65
	ONT 48h	7729	184206	1.42	11.4
	IT	229.6	1879116		
Kp_03	ONT 20h	8146	108000	0.879	12
	ONT 48h	7948	146906	1.16	10.8
	IT	192.3	2356684		
Kp_04	ONT 20h	7312	60000	0.438	11.9
	ONT 48h	7059	86546	0.61	10.6

	IT	186.6	1919262		
Kp_05	ONT 20h	7584	232000	1.76	12
	ONT 48h	7402	308658	2.28	10.8
	IT	186.6	2435748		
Kp_06	ONT 20h	4967	96000	0.476	11.8
	ONT 48h	4925	137165	0.675	10.5
	IT	196.5	1718698		

Table S4. Information on Genome Assembly. Assembled genome size, number of contigs, largest contig size, GC content percentage (%), N50, L50, coverage, Average Nucleotide Identity (ANI) and Alignment Fraction (AF) for the genomes of each bacterial isolate obtained using different sequencing/assembly protocols. ANI and AF were calculated using the tool FastANI v.1.34 (Jain et al. Nat Commun. 2018. 9(1):5114. doi: 10.1038/s41467-018-07641-9).

Isolate	Protocol	Genome size (bp)	N contigs	Maior contig (bp)	GC (%)	N50	L50	Coverage	ANI	AF
MRSA_01	ONT20h	2973236	3	2894099	32.75	2894099	1	451	98.89	90.2020202
	ONT48hA	3005125	19	2919818	32.72	2919818	1	712	98.83	87.1219512
	ONT48hB	2973234	3	2894095	32.75	2894095	1	672	98.85	90.2020202
	IT	2940227	184	384588	32.66	132375	14	90	98.84	46.1823966
	Hybrid	2916956	6	2790827	32.78	2790827	1		98.86	92.2680412
MRSA_02	ONT20h	2956724	3	2890938	32.79	2890938	1	140	98.91	90.9552846
	ONT48hA	3019190	10	2891349	32.79	2891349	1	270	98.61	89.8203593
	ONT48hB	2956626	3	2890982	32.79	2890982	1	241	98.8	91.2601626
	IT	2931855	72	649475	32.67	291262	7	50	98.82	46.4994775
	Hybrid	2956471	8	2858412	32.79	2858412	1		98.84	91.3353721
MRSA_03	ONT20h	2956981	3	2891212	32.78	2891212	1	203	98.85	91.1585366
	ONT48hA	2960014	2	2905725	32.79	2905725	1	304	98.73	90.3651116
	ONT48hB	2984308	3	2891229	32.73	2891229	1	266	98.85	90.0302115
	IT	2934750	75	682262	32.67	291262	7	62	98.88	46.2140992
	Hybrid	2958384	4	2891204	32.78	2891204	1		98.91	91.2601626
MRSA_04	ONT20h	2880147	1	2880147	32.87	2880147	1	137	98.88	92.7083333
	ONT48hA	3055526	52	2880904	32.88	2880904	1	198	98.8	90.3846154
	ONT48hB	2880252	1	2880252	32.87	2880252	1	200	98.87	92.9166667
	IT	2859106	60	118004	32.80	47762	41	56	98.86	47.3047305
	Hybrid	2879684	2	2878320	32.87	2878320	1		98.92	93.1178311
MRSA_05	ONT20h	2888247	2	2860868	32.82	2860868	1	135	98.91	92.3076923
	ONT48hA	2909778	3	2853521	32.83	2853521	1	247	98.7	92.0454545
	ONT48hB	2888288	2	2860909	32.82	2860909	1	232	98.87	93.035343
	IT	2903284	234	215338	32.75	94681	22	115	98.84	46.9983775
	Hybrid	2888473	2	2861082	32.82	2861082	1		98.88	93.1392931
MRSA_06	ONT20h	2890049	2	2862670	32.82	2862670	1	247	98.89	93.0425753
	ONT48hA	2941192	6	2869625	32.84	2869625	1	492	98.67	91.4110429
	ONT48hB	2917467	2	2862708	32.77	2862708	1	436	98.84	92.2839506
	IT	2873270	75	998437	32.71	618288	4	126	98.93	47.1056824
	Hybrid	2891663	3	2862908	32.82	2862908	1		98.91	92.7310488
MRSA_07	ONT20h	2890310	2	2862919	32.82	2862919	1	296	98.86	93.2502596
	ONT48hA	2952337	7	2880192	32.80	2880192	1	421	98.8	91.2334353
	ONT48hB	2890289	2	2862897	32.82	2862897	1	399	98.89	93.0425753
	IT	2929472	337	178675	32.73	59043	30	116	98.83	46.978022
	Hybrid	2891667	3	2862912	32.82	2862912	1		98.91	92.7310488
MRSA_08	ONT20h	2917394	2	2862658	32.77	2862658	1	325	98.85	91.872428
	ONT48hA	2943708	3	2887456	32.82	2887456	1	599	98.72	91.4198161
	ONT48hB	2917466	2	2862704	32.77	2862704	1	579	98.88	91.7695473

	IT	2900373	214	246773	32.70	77935	24	85	98.86	46.9598263
	Hybrid	2890314	2	2862923	32.82	2862923	1		98.91	92.834891
MRSA_09	ONT20h	2880026	1	2880026	32.87	2880026	1	772	98.88	93.0208333
	ONT48hA	2951137	9	2917972	32.89	2917972	1	1443	98.69	91.8200409
	ONT48hB	2880072	1	2880072	32.87	2880072	1	1429	98.87	92.8125
	IT	2902220	271	164139	32.77	73991	25	100	98.92	46.8423943
	Hybrid	2881604	2	2880240	32.87	2880240	1		98.9	93.3333333
MRSA_10	ONT20h	2864901	2	2837506	32.79	2837506	1	346	98.92	93.3962264
	ONT48hA	2909215	2	2854668	32.75	2854668	1	547	98.81	91.9504644
	ONT48hB	2864791	2	2837396	32.78	2837396	1	523	98.96	92.9769392
	IT	2845359	82	749973	32.66	211267	7	59	98.94	47.1190092
	Hybrid	2864744	2	2837353	32.78	2837353	1		98.96	92.9769392
MRSA_11	ONT20h	2864451	2	2837072	32.79	2837072	1	120	98.95	92.6624738
	ONT48hA	2901754	3	2845636	32.79	2845636	1	232	98.76	91.8219462
	ONT48hB	2864508	2	2837128	32.78	2837128	1	214	98.89	93.3962264
	IT	2849048	81	749843	32.68	304963	5	130	98.97	47.0461869
	Hybrid	2864740	2	2837349	32.78	2837349	1		98.96	92.9769392
MRSA_12	ONT20h	2868102	3	2838960	32.78	2838960	1	157	98.92	93.4031414
	ONT48hA	3000655	5	2834333	32.56	2834333	1	231	98.84	89.0781563
	ONT48hB	2893757	2	2838957	32.73	2838957	1	226	98.93	92.0124481
	IT	2846383	78	831076	32.65	283474	6	99	99	46.9860065
	Hybrid	2865928	2	2838528	32.78	2838528	1		98.94	92.9842932
MRSA_13	ONT20h	2832763	2	2795580	32.81	2795580	1	345	98.14	92.3647932
	ONT48hA	2940613	2	2836262	32.78	2836262	1	444	98.09	88.7640449
	ONT48hB	2869956	2	2795581	32.78	2795581	1	420	98.19	90.6806283
	IT	2845650	360	329005	32.80	127544	13	119	98.21	46.7475193
	Hybrid	2835913	3	2795359	32.80	2795359	1		98.15	91.8432203
MRSA_14	ONT20h	2837691	3	2801525	32.79	2801525	1	142	98.25	92.9025424
	ONT48hA	2886478	2	2819281	32.80	2819281	1	167	98.11	91.2591051
	ONT48hB	2837402	3	2801499	32.79	2801499	1	163	98.26	92.690678
	IT	2861581	421	127572	32.75	43162	43	105	98.31	47.3260495
	Hybrid	2838459	2	2801535	32.79	2801535	1		98.26	92.8042328
MRSA_15	ONT20h	2837976	3	2801816	32.81	2801816	1	115	98.19	92.9025424
	ONT48hA	2848059	4	2448103	32.87	2448103	1	166	98.13	91.5611814
	ONT48hB	2837991	3	2801833	32.81	2801833	1	157	98.29	92.4788136
	IT	2870257	615	174067	32.86	76731	27	108	98.22	47.31243
	Hybrid	2839396	2	2800987	32.81	2800987	1		98.15	93.015873
MRSA_16	ONT20h	2881599	1	2881599	32.87	2881599	1	946	98.23	91.3541667
	ONT48hA	2991005	4	2942103	32.82	2942103	1	1787	98.09	88.040201
	ONT48hB	2881594	1	2881594	32.87	2881594	1	804	98.15	91.3541667
	IT	2893922	230	176739	32.79	65424	29	127	98.24	47.1897607
	Hybrid	2860309	1	2860309	32.86	2860309	1		98.22	91.7103882
MRSA_17	ONT20h	2899498	4	2868485	32.83	2868485	1	500	98.22	91.3989637
	ONT48hA	3103000	4	2923992	32.68	2923992	1	620	98.1	85.8527132
	ONT48hB	2899427	4	2868469	32.83	2868469	1	283	98.22	91.6062176
	IT	2884549	385	196420	32.91	73501	30	79	98.18	47.1625344
	Hybrid	2888572	2	2854867	32.82	2854867	1		98.18	92.4116424

MRSA_18	ONT20h	2938146	2	2870740	32.77	2870740	1	466	98.19	90.4907975
	ONT48hA	3068200	4	2934942	32.76	2934942	1	758	98.12	86.1900098
	ONT48hB	2938174	2	2870756	32.77	2870756	1	568	98.19	90.4907975
	IT	2949312	234	280564	32.73	74320	23	98	98.25	47.2267985
	Hybrid	2903083	3	2868335	32.82	2868335	1		98.18	92.2440538
MRSA_19	ONT20h	2937772	3	2892636	32.84	2892636	1	295	98.15	90.1840491
	ONT48hA	3020647	55	2921708	32.51	2921708	1	562	98.09	79.2196007
	ONT48hB	2938174	2	2870756	32.77	2870756	1	559	98.11	89.5643364
	IT	2979324	540	243608	32.82	48200	33	123	98.2	49.0283176
	Hybrid	2934129	3	2892532	32.85	2892532	1		98.19	89.457523
MRSA_20	ONT20h	2786273	1	2786273	32.88	2786273	1	273	98.13	94.0732759
	ONT48hA	2814962	1	2814962	32.92	2814962	1	418	98.06	92.4307036
	ONT48hB	2786293	1	2786293	32.88	2786293	1	404	98.23	92.6724138
	IT	2785496	237	244755	32.79	67817	28	82	98.2	47.4431818
	Hybrid	2789113	2	2786061	32.87	2786061	1		98.22	92.8955867
MRSA_21	ONT20h	2836026	1	2836026	32.85	2836026	1	431	98.2	92.9100529
	ONT48hA	2859214	1	2859214	32.89	2859214	1	653	98.1	92.7597062
	ONT48hB	2836058	1	2836058	32.85	2836058	1	637	98.2	93.2275132
	IT	2866481	391	124043	32.78	39436	43	120	98.21	47.766323
	Hybrid	2835971	1	2835971	32.85	2835971	1		98.21	92.4867725
MRSA_22	ONT20h	2812733	2	2762615	32.79	2762615	1	255	99.83	96.5811966
	ONT48hA	2843582	2	2793744	32.81	2793744	1	313	99.74	95.0369588
	ONT48hB	2812750	2	2762630	32.79	2762630	1	299	99.83	96.9017094
	IT	2757637	50	1062250	32.71	290108	6	140	99.9	49.0628445
	Hybrid	2787669	2	2762609	32.82	2762609	1		99.86	97.1982759
MRSA_23	ONT20h	2785883	2	2760824	32.82	2760824	1	219	99.83	97.6293103
	ONT48hA	2829556	2	2780525	32.80	2780525	1	260	99.79	95.6475584
	ONT48hB	2810939	2	2760822	32.79	2760822	1	248	99.86	96.5811966
	IT	2756531	47	1062513	32.71	598499	4	117	99.91	49.0358127
	Hybrid	2785870	2	2760810	32.82	2760810	1		99.89	96.875
MRSA_24	ONT20h	2806911	1	2806911	32.90	2806911	1	237	99.81	97.4331551
	ONT48hA	2988939	23	2863972	32.76	2863972	1	336	99.66	92.9735234
	ONT48hB	2806903	1	2806903	32.90	2806903	1	212	99.83	97.1122995
	IT	2780671	48	804685	32.78	725170	4	137	99.83	49.2072171
	Hybrid	2809316	2	2806918	32.89	2806918	1		99.78	97.4331551
MRSA_25	ONT20h	2832022	2	2806961	32.86	2806961	1	253	99.77	97.0307529
	ONT48hA	2928320	6	2844203	32.81	2844203	1	369	99.69	93.7307297
	ONT48hB	2832020	2	2806958	32.86	2806958	1	343	99.8	96.6065748
	IT	2807739	52	804684	32.76	725170	4	108	99.83	48.7262873
	Hybrid	2834429	3	2806970	32.85	2806970	1		99.8	96.6065748
MRSA_26	ONT20h	2806985	1	2806985	32.90	2806985	1	523	99.8	97.4331551
	ONT48hA	2959652	6	2869343	32.82	2869343	1	717	99.7	92.1747967
	ONT48hB	2806977	1	2806977	32.90	2806977	1	739	99.76	97.8609626
	IT	2805574	50	780700	32.74	725170	4	125	99.78	48.6984816
	Hybrid	2834431	3	2806972	32.85	2806972	1		99.8	96.6065748
MRS[illegible]	ONT20h	2802092	1	2802092	32.89	2802092	1	451	99.8	98.1798715
	ONT48hA	2839227	2	2835541	32.92	2835541	1	601	99.68	96.9344609

	ONT48hB	2802085	1	2802085	32.89	2802085	1	608	99.78	98.2869379
	IT	2776401	43	1044241	32.78	718613	4	98	99.83	49.4805905
	Hybrid	2802097	1	2802097	32.88	2802097	1		99.81	98.2869379
MRSA_28	ONT20h	2817167	1	2817167	32.88	2817167	1	464	99.84	97.5505857
	ONT48hA	2910001	3	2858715	32.96	2858715	1	640	99.71	94.4214876
	ONT48hB	2817160	1	2817160	32.88	2817160	1	640	99.84	97.5505857
	IT	2854218	372	161401	32.83	52640	36	113	99.79	49.1766042
	Hybrid	2817165	1	2817165	32.88	2817165	1		99.8	97.8700745
MRSA_29	ONT20h	2854691	3	2799352	32.84	2799352	1	513	99.75	96.5263158
	ONT48hA	2920725	7	2831418	32.81	2831418	1	927	99.72	94.0206186
	ONT48hB	2799362	1	2799362	32.89	2799362	1	800	99.82	97.7491961
	IT	2884866	474	122452	32.79	38590	47	100	99.82	49.132948
	Hybrid	2826800	3	2799343	32.85	2799343	1		99.82	97.2369819
MRSA_30	ONT20h	2824431	2	2799370	32.85	2799370	1	405	99.74	97.3432519
	ONT48hA	2937322	8	2833088	32.78	2833088	1	729	99.7	93.6344969
	ONT48hB	2799365	1	2799365	32.89	2799365	1	615	99.82	97.7491961
	IT	2799832	50	1059946	32.74	601153	4	110	99.8	49.1830065
	Hybrid	2826801	3	2799344	32.85	2799344	1		99.82	97.2369819
MRSA_31	ONT20h	2782358	1	2782358	32.87	2782358	1	591	99.87	97.4110032
	ONT48hA	2886151	4	2824249	32.83	2824249	1	983	99.76	94.2708333
	ONT48hB	2782335	1	2782335	32.87	2782335	1	930	99.87	97.5188781
	IT	2865776	475	140605	32.73	38717	42	90	99.86	48.7238979
	Hybrid	2809436	2	2782170	32.81	2782170	1		99.87	96.5811966
MRSA_32	ONT20h	2788511	2	2761245	32.81	2761245	1	169	99.88	96.8783638
	ONT48hA	2825140	3	2766682	32.78	2766682	1	273	99.79	95.8510638
	ONT48hB	2815761	2	2761227	32.75	2761227	1	252	99.89	96.0554371
	IT	2765189	47	766322	32.71	630294	4	142	99.9	48.9303346
	Hybrid	2788497	2	2761231	32.81	2761231	1		99.88	97.0936491
MRSA_33	ONT20h	2863301	2	2836028	32.81	2836028	1	117	99.8	95.0733753
	ONT48hA	2988592	5	2851195	32.63	2851195	1	139	99.69	90.9547739
	ONT48hB	2890566	2	2836022	32.76	2836022	1	138	99.79	94.6002077
	IT	2835842	52	811118	32.70	421833	5	138	99.85	47.9099678
	Hybrid	2863231	2	2835960	32.81	2835960	1		99.79	95.3878407
MRSA_34	ONT20h	2822120	1	2822120	32.87	2822120	1	448	99.81	96.3829787
	ONT48hA	2941402	3	2881148	32.79	2881148	1	718	99.67	92.6455567
	ONT48hB	2821967	1	2821967	32.87	2821967	1	651	99.81	96.5957447
	IT	2824850	61	811583	32.71	250038	6	145	99.83	48.3037157
	Hybrid	2848877	4	2624440	32.82	2624440	1		99.8	95.5696203
MRSA_35	ONT20h	2845621	2	2818359	32.80	2818359	1	404	99.75	97.0464135
	ONT48hA	2952076	4	2865241	32.74	2865241	1	753	99.68	93.4893184
	ONT48hB	2845624	2	2818356	32.80	2818356	1	730	99.81	96.835443
	IT	2889928	400	164302	32.73	53303	39	125	99.82	48.6348123
	Hybrid	2845561	2	2818296	32.80	2818296	1		99.76	97.0464135
MRSA_3	ONT20h	2845609	2	2818344	32.80	2818344	1	415	99.79	96.5189873
	ONT48hA	3004038	8	2850926	32.64	2850926	1	780	99.74	91.5831663
	ONT48hB	2872882	2	2818349	32.75	2818349	1	771	99.79	95.6112853
	IT	2880300	369	157452	32.72	52166	34	109	99.82	48.6790332

	Hybrid	2845559	2	2818294	32.80	2818294	1		99.76	97.0464135
MRSA_37	ONT20h	2859500	2	2805001	32.76	2805001	1	366	99.82	95.90766
	ONT48hA	2926857	4	2840640	32.72	2840640	1	660	99.65	94.4501542
	ONT48hB	2805018	1	2805018	32.87	2805018	1	652	99.79	97.9679144
	IT	2818343	246	319847	32.71	117904	15	80	99.8	49.0628445
	Hybrid	2832221	2	2804956	32.81	2804956	1		99.77	97.1367975
MRSA_38	ONT20h	2845598	2	2818333	32.80	2818333	1	365	99.75	97.1518987
	ONT48hA	2948097	4	2857059	32.70	2857059	1	672	99.69	93.4760449
	ONT48hB	2845604	2	2818341	32.80	2818341	1	664	99.78	96.9409283
	IT	2849014	220	205805	32.70	59017	30	81	99.81	48.7804878
	Hybrid	2845550	2	2818285	32.80	2818285	1		99.76	97.0464135
MRSA_39	ONT20h	2815927	1	2815927	32.83	2815927	1	274	98.93	94.45629
	ONT48hA	2862846	7	2832411	32.83	2832411	1	386	98.86	92.6470588
	ONT48hB	2815934	1	2815934	32.83	2815934	1	372	98.92	94.3496802
	IT	2965740	934	69330	32.79	18017	101	109	98.96	47.9750779
	Hybrid	2819158	2	2815826	32.82	2815826	1		98.97	94.2492013
MRSA_40	ONT20h	2831485	1	2831485	32.84	2831485	1	540	98.96	93.0010604
	ONT48hA	3043106	35	2860549	32.69	2860549	1	776	98.87	87.9120879
	ONT48hB	2831458	1	2831458	32.84	2831458	1	795	98.93	94.0615058
	IT	2803588	172	337224	32.73	96949	16	93	98.99	47.5630252
	Hybrid	2829658	2	2826326	32.84	2826326	1		98.98	93.2131495
MRSA_41	ONT20h	2818510	1	2818510	32.82	2818510	1	464	98.95	94.0362087
	ONT48hA	3002206	72	2843634	32.49	2843634	1	657	98.86	85.4247104
	ONT48hB	2818481	1	2818481	32.82	2818481	1	710	98.92	94.6751864
	IT	2785779	124	269641	32.71	76417	22	89	98.99	47.8334271
	Hybrid	2824786	3	2818010	32.81	2818010	1		98.87	94.3676939
MRSA_42	ONT20h	2829428	4	2825710	32.83	2825710	1	147	98.91	94.0488842
	ONT48hA	3030220	49	2836203	32.65	2836203	1	186	98.86	88.7662989
	ONT48hB	2825663	1	2825663	32.84	2825663	1	193	98.94	93.9426142
	IT	2821665	218	385103	32.79	171275	10	143	99	47.6948408
	Hybrid	2828240	3	2713309	32.83	2713309	1		98.9	94.1613588
Kp_01	ONT20h	5497569	2	5275553	57.24	5275553	1	71	99.92	93.941048
	ONT48hB	5497931	2	5275899	57.24	5275899	1	96	99.92	94.0502183
	IT	5117306	1	5117306	57.70	5117306	1	86	99.89	92.4340176
Kp_02	ONT20h	5588684	2	5321936	57.14	5321936	1	170	99.81	92.2622246
	ONT48hB	5497936	2	5275916	57.24	5275916	1	255	99.92	94.0502183
	IT	5115219	1	5115219	57.73	5115219	1	78	99.91	90.2052786
Kp_03	ONT20h	5352345	3	5213720	57.43	5213720	1	161	99.02	91.3580247
	ONT48hB	5338690	2	5213945	57.45	5213945	1	206	99.03	91.3385827
	IT	5072626	1	5072626	58.10	5072626	1	85	99.03	83.4911243
Kp_04	ONT20h	5351997	3	5213393	57.43	5213393	1	80	99.01	91.4141414
	ONT48hB	5338190	2	5213441	57.45	5213441	1	108	98.99	91.4510686
	IT	5096305	1	5096305	57.92	5096305	1	67	99.04	89.5170789
Kp_05	ONT20h	5352323	3	5213700	57.43	5213700	1	320	99.04	91.2457912
	ONT48hB	5338387	2	5213632	57.45	5213632	1	401	99.02	91.3385827
	IT	5096509	1	5096509	57.88	5096509	1	85	99.07	91.1071849
[illegible]	ONT20h	5351922	3	5213370	57.43	5213370	1	86	99.01	91.3580247

	ONT48hB	5338231	2	5213475	57.45	5213475	1	118	99.01	91.2823397
	IT	5062603	1	5062603	57.92	5062603	1	63	99.03	89.5080024

Table S5. Table of results from genomic analyses conducted using the ResFinder service with MRSA genomes obtained through the ONT20h protocol.

**ISOLATE**	**GENE**										
	mecA	*blaZ*	*tet(M)*	*lnu(A)*	*tet(K)*	*aac(6')-aph(2'')*	*msr(A)*	*mph(C)*	*dfrG*	*ant(6)-Ia*	*aph(3')-III*
MRSA_01	A	A	B								
MRSA_02	B	B	B								
MRSA_03	A	A	B								
MRSA_04	A		B								
MRSA_05	A	A	B								
MRSA_06	C	A	C								
MRSA_07	A	A	B								
MRSA_08	A	A	C								
MRSA_09	B		B								
MRSA_10	A	A	B								
MRSA_11	A	A	C								
MRSA_12	A	A	B								
MRSA_13	A	B									
MRSA_14	A	B		B							
MRSA_15	A	B									
MRSA_16	A	B									
MRSA_17	A	A		B							
MRSA_18	A	A		B							
MRSA_19	A	B			B						
MRSA_20	A			B							
MRSA_21	A	B		B							
MRSA_22	A	A									
MRSA_23	A	A									
MRSA_24	A										
MRSA_25	A	A									
MRSA_26	A										
MRSA_27	A										
MRSA_28	A										
MRSA_29	A	A									
MRSA_30	A	A									
MRSA_31	A					A					
MRSA_32	A	A				A					
MRSA_33	A	A									
MRSA_34	A										
MRSA_35	A	A									
MRSA_36	A	A									
MRSA_37	A	A									
MRSA_38	A	A									
MRSA_39	A	A				A	C	A	A	C	A
MRSA_40	A	A				A	C	A	A	C	A
MRSA_41	A	A				A	B	A	A	C	A
MRSA_42	A	A				A	C	A	A	C	A

A Category A (dark green): Gene Size in the Input Genome = Reference Gene Size. Identity = 100%
B Category B (light green): Gene Size in the Input Genome = Reference Gene Size. Identity < 100%
C Category C (gray): Gene Size in the Input Genome ≠ Reference Gene Size. Identity ≤ 100%

Table S6. Table of results from genomic analyses conducted using the ResFinder service with MRSA genomes obtained through the ONT48hA protocol.

ISOLATE	GENE										
	mecA	*blaZ*	*tet(M)*	*lnu(A)*	*tet(K)*	*aac(6')-aph(2'')*	*msr(A)*	*mph(C)*	*dfrG*	*ant(6)-Ia*	*aph(3')-III*
MRSA_01	B	B	B								
MRSA_02	B	B	B								
MRSA_03	B	B	B								
MRSA_04	B		B								
MRSA_05	B	B	B								
MRSA_06	B	B	B								
MRSA_07	B	B	B								
MRSA_08	B	B	B								
MRSA_09	B		B								
MRSA_10	B	B	B								
MRSA_11	B	B	B								
MRSA_12	B	B	B								
MRSA_13	B	B									
MRSA_14	B	B		B							
MRSA_15	B	B									
MRSA_16	B	B			B						
MRSA_17	B	B		B							
MRSA_18	B	B		B							
MRSA_19	B	B			B						
MRSA_20	B			B							
MRSA_21	B	B		B							
MRSA_22	B	B									
MRSA_23	B	B									
MRSA_24	B										
MRSA_25	B	B									
MRSA_26	B	B									
MRSA_27	B										
MRSA_28	B										
MRSA_29	B	B									
MRSA_30	B	B									
MRSA_31	B	B				B					
MRSA_32	B	B				B					
MRSA_33	B	B									
MRSA_34	B	B									
MRSA_35	B	B			B						
MRSA_36	B	B			B						
MRSA_37	B	B			B						
MRSA_38	B	B			B						
MRSA_39	B	B				B	B	A	B	C	B
MRSA_40	B	B				B	B	A	B	C	B
MRSA_41	B	B				B	B	A	B	C	B
MRSA_42	B	B				B	B	B	A	C	B

A Category A (dark green): Gene Size in the Input Genome = Reference Gene Size. Identity = 100%

B Category B (light green): Gene Size in the Input Genome = Reference Gene Size. Identity < 100%

C Category C (gray): Gene Size in the Input Genome ≠ Reference Gene Size. Identity ≤ 100%

Table S7. Table of results from genomic analyses conducted using the ResFinder service with MRSA genomes obtained through the ONT48hB protocol.

**ISOLATE**	**GENE**									
	mecA	*blaZ*	*tet(M)*	*lnu(A)*	*aac(6')-aph(2'')*	*msr(A)*	*mph(C)*	*dfrG*	*ant(6)-Ia*	*aph(3')-III*
MRSA_01	A	A	B							
MRSA_02	A	B	B							
MRSA_03	A	A	B							
MRSA_04	A		B							
MRSA_05	B	A	C							
MRSA_06	A	A	C							
MRSA_07	A	A	B							
MRSA_08	A	B	C							
MRSA_09	B		B							
MRSA_10	A	A	C							
MRSA_11	A	A	C							
MRSA_12	A	A	B							
MRSA_13	A	B								
MRSA_14	A	B		B						
MRSA_15	A	B								
MRSA_16	A	B								
MRSA_17	A	A		B						
MRSA_18	A	A		B						
MRSA_19	A	B								
MRSA_20	A			B						
MRSA_21	A	B		B						
MRSA_22	A	A								
MRSA_23	A	A								
MRSA_24	A									
MRSA_25	A	A								
MRSA_26	A									
MRSA_27	A									
MRSA_28	A									
MRSA_29	A									
MRSA_30	A									
MRSA_31	A				A					
MRSA_32	A	A			A					
MRSA_33	A	A								
MRSA_34	A									
MRSA_35	A	A								
MRSA_36	A	A								
MRSA_37	A									
MRSA_38	A	A								
MRSA_39	A	A			A	C	A	A	C	A
MRSA_40	A	A			A	C	A	A	C	A
MRSA_41	A	A			A	B	A	A	C	A
MRSA_42	A	A			A	B	A	A	C	A

A	Category A (dark green): Gene Size in the Input Genome = Reference Gene Size. Identity = 100%
B	Category B (light green): Gene Size in the Input Genome = Reference Gene Size. Identity < 100%
C	Category C (gray): Gene Size in the Input Genome ≠ Reference Gene Size. Identity ≤ 100%

Table S8. Table of results from genomic analyses conducted using the ResFinder service with MRSA genomes obtained through the IT protocol.

ISOLATE	GENE										
	mecA	*blaZ*	*tet(M)*	*lnu(A)*	*tet(K)*	*aac(6')-aph(2'')*	*msr(A)*	*mph(C)*	*dfrG*	*ant(6)-Ia*	*aph(3')-III*
MRSA_01	A	A	B								
MRSA_02	A	A	B								
MRSA_03	A	A	B								
MRSA_04	A		B								
MRSA_05	A	A	C								
MRSA_06	A	A	B								
MRSA_07	A	A	B								
MRSA_08	A	A	B								
MRSA_09	A		B								
MRSA_10	A	A	B								
MRSA_11	A	A	B								
MRSA_12	A	A	B								
MRSA_13	A	B									
MRSA_14	A	B		B							
MRSA_15	A	B									
MRSA_16	A	B									
MRSA_17	A	A		B							
MRSA_18	A	A		B							
MRSA_19	A	B			A						
MRSA_20	A			B							
MRSA_21	A	B		B							
MRSA_22	A	A									
MRSA_23	A	A									
MRSA_24	A										
MRSA_25	A	A									
MRSA_26	A	A									
MRSA_27	A										
MRSA_28	A										
MRSA_29	A	A									
MRSA_30	A	A									
MRSA_31	A	A				A					
MRSA_32	A	A				A					
MRSA_33	A	A									
MRSA_34	A	A									
MRSA_35	A	A									
MRSA_36	A	A									
MRSA_37	A	A									
MRSA_38	A	A									
MRSA_39	A	A				A	B	A	A	C	A
MRSA_40	A	C				A	B	A	A	C	A
MRSA_41	A	B				A	B	A	A	C	A
MRSA_42	A	A				A	B	A	A	C	A

A Category A (dark green): Gene Size in the Input Genome = Reference Gene Size. Identity = 100%
B Category B (light green): Gene Size in the Input Genome = Reference Gene Size. Identity < 100%
C Category C (gray): Gene Size in the Input Genome ≠ Reference Gene Size. Identity ≤ 100%

Table S9. Table of results from genomic analyses conducted using the ResFinder service with MRSA genomes obtained through the Hybrid protocol.

ISOLATE	GENE											
	mecA	*blaZ*	*tet(M)*	*lnu(A)*	*tet(K)*	*aac(6')-aph(2'')*	*msr(A)*	*mph(C)*	*dfrG*	*ant(6)-Ia*	*aph(3')-III*	*aph(2'')-Ia*
MRSA_01	A	A	B									
MRSA_02	A	A	B									
MRSA_03	A	A	B									
MRSA_04	A		B									
MRSA_05	A	A	B									
MRSA_06	A	A	B									
MRSA_07	A	A	B									
MRSA_08	A	A	B									
MRSA_09	A		B									
MRSA_10	A	A	B									
MRSA_11	A	A	B									
MRSA_12	A	A	B									
MRSA_13	A	B										
MRSA_14	A	B		B								
MRSA_15	A	B										
MRSA_16	A	B										
MRSA_17	A	A		B								
MRSA_18	A	A		B								
MRSA_19	A	B			A							
MRSA_20	A			B								
MRSA_21	A	B		B								
MRSA_22	A	A										
MRSA_23	A	A										
MRSA_24	A											
MRSA_25	A	A										
MRSA_26	A	A										
MRSA_27	A											
MRSA_28	A											
MRSA_29	A	A										
MRSA_30	A	A										
MRSA_31	A	A				A						
MRSA_32	A	A				A						
MRSA_33	A	A										
MRSA_34	A	A										
MRSA_35	A	A										
MRSA_36	A	A										
MRSA_37	A	A										
MRSA_38	A	A										
MRSA_39	A	A				C	B	A	A	C	A	C
MRSA_40	A	A					B	A	A	C	A	
MRSA_41	A	B				C	B	A		C	A	
MRSA_42	A	A				A	B	A	A	C	A	

A Category A (dark green): Gene Size in the Input Genome = Reference Gene Size. Identity = 100%

B Category B (light green): Gene Size in the Input Genome = Reference Gene Size. Identity < 100%

C Category C (gray): Gene Size in the Input Genome ≠ Reference Gene Size. Identity ≤ 100%

Table S10. Table of results from genomic analyses conducted using the CARD-RGI service with MRSA genomes obtained through the ONT20h protocol.

ISOLATE	GENE																						
	arlR	*arlS*	*dfrC*	*mecA*	*mepA*	*mepR*	*mgrA*	*blaZ*	*fosB*	*LmrS*	*murA*	*norA*	*tet(W/N/W)*	*tet(M)*	*lnuA*	*fusA*	*AAC(6')-Ie-APH(2'')-Ia*	*gyrA*	*parC*	*APH(3')-IIIa*	*mphC*	*dfrG*	*msrA*
MRSA_01	A	A	B	B	A	A	A	B	A	A	B	B	C										
MRSA_02	A	A			A	A	A		A	A	B	B	C										
MRSA_03	A	A		B	A	A	A	B	A	A	B	B	C										
MRSA_04	A	A	B	B	A	A	A		A	A	B	B		B									
MRSA_05	A	A	B	B	A	A	A	B	A	A	B	B	C										
MRSA_06	A	A	B		A	A	A	B	A	A	B	B											
MRSA_07	A	A	B	B	A	A	A	B	A	A	B	B	C										
MRSA_08	A	A	B	B	A	A	A	B	A	A	B	B											
MRSA_09	A	A	B		A	A	A		A	A	B	B	C										
MRSA_10	A	A	B	B	A	A	A	B	A	A	B	B	C										
MRSA_11	A	A	B	B	A	A	A	B	A	A	B	B	C										
MRSA_12	A	A	B	B	A	A	A	B	A	C	B	B	C										
MRSA_13	A	A		B		A		B		B		B											
MRSA_14	A	A		B		A	A	B		C		B			B								
MRSA_15	A		B	B		A	A	B		C		B											
MRSA_16	A	A	B	B		A	A	B		B		B											
MRSA_17	A			B		A	A	B		C		B			B								
MRSA_18	A	A		B		A	A	B		B		B			B								
MRSA_19	A	A	B	B		A	A	B		C		B											
MRSA_20	A	A		B		A	A			B		B			B								
MRSA_21	A	A		B		A	A	B		B		B			B								
MRSA_22	A	A		B		A	A	A	A	B		A											
MRSA_23	A	A		B		A	A	A	A	B		A											
MRSA_24	A	A	B	B		A	A		A	B		A											
MRSA_25	A	A	B	B		A	A	A	A	B		A											
MRSA_26	A		B	B		A	A		A	B		A											
MRSA_27	A	A		B		A	A		A	B		A				B							
MRSA_28	A	A		B		A	A		A	B		A				B							
MRSA_29	A	A		B		A	A	A	A	B		A											
MRSA_30	A	A		B		A	A	A	A	B		A											
MRSA_31	A	A		B		A	A		A	B		A					A						
MRSA_32	A	A		B		A	A	B	A	B		A					A						
MRSA_33	A	A		B		A	A	B	A	B		A											
MRSA_34	A			B		A	A		A	B		A											
MRSA_35	A	A		B		A	A	B	A	B		A						C	B				
MRSA_36	A	A		B		A	A	B	A	B		A						C	B				
MRSA_37	A	A		B		A	A	B	A	B		A						C	B				
MRSA_38	A	A		B		A	A	B	A	B		A						C	B				
MRSA_39	A	A		B		A	A	B	A	B		B					A		B	A	B	A	
MRSA_40	A	A		B		A	A	B	A	B		B					A	C	B	A	B	A	
MRSA_41	A	A		B		A	A	B	A	B		B					A	C	B	A	B	A	B
MRSA_42	A	A		B		A	A	B	A	C		B					A	C	B	A	B	A	

A	Category A (dark green): Gene Size in the Input Genome = Reference Gene Size. Identity = 100%
B	Category B (light green): Gene Size in the Input Genome = Reference Gene Size. Identity < 100%
C	Category C (gray): Gene Size in the Input Genome ≠ Reference Gene Size. Identity ≤ 100%

Table S11. Table of results from genomic analyses conducted using the CARD-RGI service with MRSA genomes obtained through the ONT48hA protocol.

ISOLATE	GENE												
	arlR	*mepA*	*mepR*	*mgrA*	*fosB*	*LmrS*	*murA*	*norA*	*fusA*	*gyrA*	*parC*	*mphC*	*dfrG*
MRSA_01	A	A	A	A		C	B						
MRSA_02	A	A	A	A		C	B						
MRSA_03	A	A	A	A		A	B						
MRSA_04	A	A	A	A		C	B						
MRSA_05	A	A	A	A		C	B						
MRSA_06	A	A	A	A		C	B						
MRSA_07	A	A	A	A		C	B	C					
MRSA_08	A	A	A	A		C	B						
MRSA_09	A	A	A	A		C	C						
MRSA_10	A	A	A	A		A	B						
MRSA_11	A	A	A	A		C	B						
MRSA_12	A	A	A	A		C	B						
MRSA_13	A		A	A		B							
MRSA_14	A		A	A		B							
MRSA_15	A		A	A		C							
MRSA_16	A		A	A		C							
MRSA_17	A		A	A		C							
MRSA_18	A		A	A		B							
MRSA_19	A		A	A		C		C					
MRSA_20	A		A	A		B							
MRSA_21	A		A	A		B							
MRSA_22	A		A	A		B		A					
MRSA_23	A		A	A		B							
MRSA_24	A		A	A		C							
MRSA_25	A		A	A		C							
MRSA_26	A		A	A		C							
MRSA_27	A		A	A		C			C				
MRSA_28	A		A	A		C			C				
MRSA_29	A		A	A	A	C							
MRSA_30	A		A	A		C		C					
MRSA_31	A		A	A	A	C		C					
MRSA_32	A		A	A	A	C							
MRSA_33	A		A	A	A	C							
MRSA_34	A		A	A		C							
MRSA_35	A		A	A		C				C	C		
MRSA_36	A		A	A	A	C				C	C		
MRSA_37	A		A	A		C		C		C	C		
MRSA_38	A		A	A		C				C	C		
MRSA_39	A		A	A		C					C	B	C
MRSA_40	A		A	A		C				C	C	B	C
MRSA_41	A		A	A	A	C				C	C	B	C
MRSA_42	A		A	A		C				C	C		A

A Category A (dark green): Gene Size in the Input Genome = Reference Gene Size. Identity = 100%

B Category B (light green): Gene Size in the Input Genome = Reference Gene Size. Identity < 100%

C Category C (gray): Gene Size in the Input Genome ≠ Reference Gene Size. Identity ≤ 100%

Table S12. Table of results from genomic analyses conducted using the CARD-RGI service with MRSA genomes obtained through the ONT48hB protocol.

ISOLATE	GENE																									
	arlR	*arlS*	*dfrC*	*mecA*	*mepA*	*mepR*	*mgrA*	*blaZ*	*fosB*	*LmrS*	*murA*	*norA*	*tet(M)*	*lnuA*	*fusA*	*AAC(6')-Ie-APH(2'')-Ia*	*gyrA*	*parC*	*APH(3')-IIIa*	*mphC*	*dfrG*	*msrA*	*sdrM*	*vanT*	*norC*	*sepA*
MRSA_01	A	A	B	B	A	A	A	B	A	A	B	B	C										A	C	B	B
MRSA_02	A	A		B	A	A	A		A	A	B	B	C										A	C	B	C
MRSA_03	A	A		B	A	A	A	B	A	A	B	B	C										A	C	B	B
MRSA_04	A	A	B	B	A	A	A		A	A	B	B	B										A	C	B	B
MRSA_05	A	A	B		A	A	A	B	A	A	B	B	C										A	C	B	B
MRSA_06	A	A	B	B	A	A	A	B	A	A	B	B	C										A	C	B	C
MRSA_07	A	A	B	B	A	A	A	B	A	A	B	B	C										A	C	B	B
MRSA_08	A	A	B	B	A	A	A		A	A	B	B	C										A		B	C
MRSA_09	A	A	B		A	A	A		A	A	B	B	C										A	C	B	B
MRSA_10	A	A	B	B	A	A	A	B	A	A	B	B	C										A	C	B	B
MRSA_11	A	A	B	B	A	A	A	B	A	A	B	B	C										A	C	B	B
MRSA_12	A	A	B	B	A	A	A	B	A	A	B	B	C										A	C	B	C
MRSA_13	A	A		B		A	B	B		B		B											B	C	B	C
MRSA_14	A	A		B		A	A	B		C		B		B									C	C	B	C
MRSA_15	A		B	B		A	A	B		C		B											B	C	B	C
MRSA_16	A	A	B	B		A	A	B		B		B											C	C	B	C
MRSA_17	A			B		A	A	B		C		B		B									C	C	B	C
MRSA_18	A	A		B		A	A	B		B		B		B									C	C	B	C
MRSA_19	A	A	B	B		A	A	B		C		B											B	C	B	C
MRSA_20	A	A		B		A	A			B		B		B									C	C	B	C
MRSA_21	A	A		B		A	A	B		B		B		B									B	C	B	C
MRSA_22	A	A		B		A	A	A	A	B		A											C	C	B	B
MRSA_23	A	A		B		A	A	A	A	B		A											C	C	B	B
MRSA_24	A	A	B	B		A	A		A	B		A											C	C	B	B
MRSA_25	A	A	B	B		A	A	A	A	B		A											C	C	B	B
MRSA_26	A		B	B		A	A		A	B		A											C	C	B	B
MRSA_27	A	A		B		A	A		A	B		A			B								C	C	B	B
MRSA_28	A	A		B		A	A		A	B		A			B								C	C	B	B
MRSA_29	A	A		B		A	A		A	B		A											C	C	B	B
MRSA_30	A	A		B		A	A		A	B		A											C	C	B	B
MRSA_31	A	A		B		A	A		A	B		A				A							A	C	B	B
MRSA_32	A	A		B		A	A	B	A	B		A				A							A	C	B	C
MRSA_33	A	A		B		A	A	B	A	B		A											B	C	B	C
MRSA_34	A			B		A	A		A	B		A											B	C	B	C
MRSA_35	A	A		B		A	A	B	A	B		A					C	B					B	C	B	B
MRSA_36	A	A		B		A	A	B	A	B		A					C	B					B	C	B	B
MRSA_37	A	A		B		A	A		A	B		A					C	B					B	C	B	B
MRSA_38	A	A		B		A	A	B	A	B		A					C	B					B	C	B	B
MRSA_39	A	A		B		A	A	B	A	B		A				A		B	A	B	A		A	C	B	B
MRSA_40	A	A		B		A	A	B	A	B		A				A	C	B	A	B	A		A	C	B	C
MRSA_41	A	A		B		A	A	B	A	B		A				A		B	A	B	A	B	A	C	B	B
MRSA_42	A	A		B		A	A	B	A	B		A				A	C	B	A	B	A	B	A	C	B	C

A	Category A (dark green): Gene Size in the Input Genome = Reference Gene Size. Identity = 100%
B	Category B (light green): Gene Size in the Input Genome = Reference Gene Size. Identity < 100%
C	Category C (gray): Gene Size in the Input Genome ≠ Reference Gene Size. Identity ≤ 100%

Table S13. Table of results from genomic analyses conducted using the CARD-RGI service with MRSA genomes obtained through the IT protocol.

ISOLATE	GENE																							
	arlR	*arlS*	*dfrC*	*mecA*	*mepA*	*mepR*	*mgrA*	*blaZ*	*fosB*	*LmrS*	*murA*	*norA*	*tet(W/N/W)*	*tet(M)*	*lnuA*	*fusA*	*AAC(6')-Ie-APH(2'')-Ia*	*gyrA*	*parC*	*APH(3')-IIIa*	*mphC*	*dfrG*	*msrA*	*tet(K)*
MRSA_01	C		C	C		C	C	C	C	C	C	C		C										
MRSA_02	C			C		C	A	C	A	C	C	B		C										
MRSA_03	A	A		C		C	C	C	A	C	C	C		C										
MRSA_04	C		C	C		C	C		C	C	C	C		C										
MRSA_05	C		C	C		C	C	C	C	C	C	C	C											
MRSA_06	C		C	C	A	A	C	B	C	C	C	C		C										
MRSA_07	C		C	C		C	C	C	C	C	C	C		C										
MRSA_08	C		C	C	A	A	C	C	C	C	C	C		C										
MRSA_09	C		C	C		C	C		C	C	C	C		C										
MRSA_10	A	A	B	B		C	C	C	A	C	C	C		C										
MRSA_11	C		B	B		C	A	C	C	C	C	C		C										
MRSA_12	A	A	C	C		C	A	C	C	C	C	B		C										
MRSA_13	C			C		C	C	C		C		C												
MRSA_14	C			C		C	C	C		C		C			C									
MRSA_15	C		C	C		C	C	C		C		C												
MRSA_16	C		C	C		C	C	C		C		C												
MRSA_17	C			C		C	C	C		C		C			C									
MRSA_18	C			C		C	C	C		C		B			C									
MRSA_19	C		C	C		C	A	C		C		C												C
MRSA_20	A	A		C		C	C			C		C			C									
MRSA_21	C			C		C	C	C		C		C			C									
MRSA_22	C			B		C	C	A	A	C		C												
MRSA_23	C			C		C	C	A	A	C		C												
MRSA_24	C		C	B		A	C		C	C		C												
MRSA_25	C		B	B		C	C	C	C	C		C												
MRSA_26	C		B	B		C	C	C	C	C		C												
MRSA_27	C			B		C	C		A	C		C				B								
MRSA_28	C			C		C	C		C	C		C				C								
MRSA_29	C			C		C	C	C	A	C		C												
MRSA_30	C			B		A	C	A	C	C		C												
MRSA_31	C			C		C	C	C		C		C					C							
MRSA_32	C			B		A	C	C	C	C		C					C							
MRSA_33	A	A		C		A	C	C	A	C		C												
MRSA_34	C			C		A	C	C	A	C		C												
MRSA_35	C			C		C	C	C	C	C		C						C	C					
MRSA_36	C			C		C	C	C	C	C		C						C	C					
MRSA_37	C			C		C	A	C		C		C						C	C					
MRSA_38	C			C		C	C	C	A	C		C						C	C					
MRSA_39	C			C		C	C	C	C	C		C					C			C	C	C	C	
MRSA_40	C			C		C	A		C	C		A					C	C		C	C	C	C	
MRSA_41	C			C		C	C	C	A	C							C	C		C	C	C	C	
MRSA_42	A	A		C		C	C	C	C								C			C	C	A	B	

Category A (dark green): Gene Size in the Input Genome = Reference Gene Size. Identity = 100%

Category B (light green): Gene Size in the Input Genome = Reference Gene Size. Identity < 100%

Category C (gray): Gene Size in the Input Genome ≠ Reference Gene Size. Identity ≤ 100%

Table S14. Table of results from genomic analyses conducted using the CARD-RGI service with MRSA genomes obtained through the Hybrid protocol.

ISOLATE	GENE																											
	arlR	*arlS*	*dfrC*	*mecA*	*mepA*	*mepR*	*mgrA*	*blaZ*	*fosB*	*LmrS*	*murA*	*norA*	*tet(M)*	*lnuA*	*fusA*	*AAC(6')-Ie-APH(2'')-Ia*	*gyrA*	*parC*	*APH(3')-IIIa*	*mphC*	*dfrG*	*msrA*	*sdrM*	*vanT*	*norC*	*sepA*	*tet(K)*	*kdpD*
MRSA_01	A	A	B	B	A	A	A	B	A	C	B	B	B										A	C	B	B		
MRSA_02	A	A		B	A	A	A	B	A	C	B	B	B										A	C	B	B		
MRSA_03	A	A		B	A	A	A	B	A	C	B	B	B										A	C	B	B		
MRSA_04	A	A	B	B	A	A	A		A	C	B	B	B										A	C	B	B		
MRSA_05	A	A	B	B	A	A	A	B	A	A	B	B	B										A	C	B	B		
MRSA_06	A	A	B	B	A	A	A	B	A	C	B	B	B										A	C	B	B		
MRSA_07	A	A	B	B	A	A	A	B	A	C	B	B	B										A	C	B	B		
MRSA_08	A	A	B	B	A	A	A	B	A	A	B	B	B										A	C	B	B		
MRSA_09	A	A	B	B	A	A	A		A	C	B	B	B										A	C	B	B		
MRSA_10	A	A	B	B	A	A	A	B	A	A	B	B	B										A	C	B	B		
MRSA_11	A	A	B	B	A	A	A	B	A	A	B	B	B										A	C	B	B		
MRSA_12	A	A	B	B	A	A	A	B	A	A	B	B	B										A	C	B	B		
MRSA_13	A	A		B		A	B	B		C		B											B	C	B	C		
MRSA_14	A	A		B		A	A	B		B		B		B									B	C	B	C		
MRSA_15	A	A	B	B		A	A	B		B		B											B	C	B	C		
MRSA_16	A	A	B	B		A	A	B		B		B											B	C	B	C		
MRSA_17	A	A		B		A	A	B		B		B		B									B	C	B	C		
MRSA_18	A	A		B		A	A	B		C		B		B									B	C	B	C		
MRSA_19	A	A	B	B		A	A	B		C		B											B	C	B	C	A	
MRSA_20	A	A		B		A	A			C		B		B									B	C	B	C		
MRSA_21	A	A		B		A	A	B		B		B		B									B	C	B	C		
MRSA_22	A	A		B		A	A	A	A	B		A											C	C	B	B		
MRSA_23	A	A		B		A	A	A	A	B		A											C	C	B	B		
MRSA_24	A	A	B	B		A	A		A	C		A											C	C	B	B		
MRSA_25	A	A	B	B		A	A	A	A	C		A											C	C	B	B		
MRSA_26	A		B	B		A	A	A	A	C		A											C	C	B	B		
MRSA_27	A	A		B		A	A		A	B		A			B								C	C	B	B		
MRSA_28	A	A		B		A	A		A	B		A			B								C	C	B	B		
MRSA_29	A	A		B		A	A	A	A	C		A											C	C	B	B		
MRSA_30	A	A		B		A	A	A	A	C		A											C	C	B	B		
MRSA_31	A	A		B		A	A	B	A	B		A				A							A	C	B	B		
MRSA_32	A	A		B		A	A	B	A	B		A				A							A	C	B	C		
MRSA_33	A	A		B		A	A	B	A	B		A											B	C	B	B		
MRSA_34	A			B		A	A	B	A	C		A											B	C	B	B		
MRSA_35	A	A		B		A	A	B	A	B		A					C	B					B	C	B	B		
MRSA_36	A	A		B		A	A	B	A	B		A					C	B					B	C	B	B		
MRSA_37	A	A		B		A	A	B	A	B		A					C	B					B	C	B	B		
MRSA_38	A	A		B		A	A	B	A	B		A					C	B					B	C	B	B		
MRSA_39	A	A		B		A	A	B	A	C		A				C		B	A	B	A	B	A	C	B	B		C
MRSA_40	A	A		B		A	A	B	A	C		A					C	B	A	B	A	B	A	C	B	B		C
MRSA_41	A	A		B		A	A		A	C		A					C	B	A	B	A	B	A	C	B	B		C
MRSA_42	A	A		B		A	A	B	A	C		A				A	C	B	A	B	A	B	A	C	B	B		C

A — Category A (dark green): Gene Size in the Input Genome = Reference Gene Size. Identity = 100%

B — Category B (light green): Gene Size in the Input Genome = Reference Gene Size. Identity < 100%

C — Category C (gray): Gene Size in the Input Genome ≠ Reference Gene Size. Identity ≤ 100%

Table S15. Table of results from genomic analyses conducted using the ResFinder service with ESBL-Kp genomes obtained through the ONT20h protocol.

ISOLATE	GENE																																
	sul1	*dfrA12*	*OqxA*	*OqxB*	*aadA2*	*aph(3')-Ia*	*aac(6')-Ib-cr*	*fosA*	*mph(A)*	*aac(6')-Ib-cr2*	*tet(A)*	*blaOXA-1*	*blaCTX-M-15*	*blaSHV-106*	*blaSHV-28*	*blaTEM-1B*	*qacE*	*catB3*	*aph(6)-Id*	*aph(3'')-Ib*	*qnrB1*	*sul2*	*dfrA14*	*blaSHV-187*	*blaTEM-206*	*blaTEM-57*	*blaTEM-216*	*blaTEM-214*	*blaTEM-210*	*blaTEM-209*	*blaTEM-34*	*blaTEM-33*	*blaTEM-141*
Kp_01	A	A	B	B	A	A	A	B	B	A	C	A	A	B	B	A	C	C															
Kp_02	A	B	B	B	B	A	A	B	B		B	B	B	B	B	B	C	C															
Kp_03			B	B				B			A		A			A			A	A	A	A	A	A									
Kp_04			B	B				B			A		B			C			A	B	A	A	A	A	C	C	C	C	C	C	C	C	C
Kp_05			B	B				B			C		A			A			A	B	A	A	A	A									
Kp_06			B	B				B			A		A			A			A	B	A	B	A	A									

Category A (dark green): Gene Size in the Input Genome = Reference Gene Size. Identity = 100%

Category B (light green): Gene Size in the Input Genome = Reference Gene Size. Identity < 100%

Category C (gray): Gene Size in the Input Genome ≠ Reference Gene Size. Identity ≤ 100%

Table S16. Table of results from genomic analyses conducted using the ResFinder service with ESBL-Kp genomes obtained through the ONT48hB protocol.

ISOLATE	GENE																																
	sul1	*dfrA12*	*OqxA*	*OqxB*	*aadA2*	*aph(3')-Ia*	*aac(6')-Ib-cr*	*fosA*	*mph(A)*	*tet(A)*	*blaOXA-1*	*blaCTX-M-15*	*blaSHV-106*	*blaSHV-28*	*blaTEM-1B*	*qacE*	*catB3*	*aph(6)-Id*	*aph(3'')-Ib*	*qnrB1*	*sul2*	*dfrA14*	*blaSHV-187*	*blaTEM-206*	*blaTEM-57*	*blaTEM-216*	*blaTEM-214*	*blaTEM-210*	*blaTEM-209*	*blaTEM-34*	*blaTEM-33*	*blaTEM-141*
Kp_01	A	A	B	B	A	A	A	B	B	C	A	A	B	B	A	C	C															
Kp_02	A	A	B	C	A	A	A	B	A	A	A	A	B	B	A	C	C															
Kp_03			B	B				B		C		A			A				A	A	A	A	A									
Kp_04			B	B				B		A		A			C			A	B	A	A	A	A	C	C	C	C	C	C	C	C	C
Kp_05			B	B				B		C		A			A			A	A	A	A	A	C									
Kp_06			B	B				B		A		A			A			A	B	A	A	A	C									

Category A (dark green): Gene Size in the Input Genome = Reference Gene Size. Identity = 100%

Category B (light green): Gene Size in the Input Genome = Reference Gene Size. Identity < 100%

Category C (gray): Gene Size in the Input Genome ≠ Reference Gene Size. Identity ≤ 100%

Table S17. Table of results from genomic analyses conducted using the ResFinder service with ESBL-Kp genomes obtained through the IT protocol.

ISOLATE	GENE					
	OqxA	*OqxB*	*fosA*	*blaSHV-106*	*blaSHV-28*	*blaSHV-187*
Kp_01	B	B	B	B	B	
Kp_02	B	B	B			A
Kp_03	B	B	B			A
Kp_04	B	B	B			A
Kp_05	B	B	B			A
Kp_06	B	B	B			A

Category A (dark green): Gene Size in the Input Genome = Reference Gene Size. Identity = 100%

Category B (light green): Gene Size in the Input Genome = Reference Gene Size. Identity < 100%

Category C (gray): Gene Size in the Input Genome ≠ Reference Gene Size. Identity ≤ 100%

Table S18. Table of results from genomic analyses conducted using the VirulenceFinder service with MRSA genomes obtained through the ONT20h protocol. All genes detected here were validated using VFDB v.5.

ISOLATE	GENE																											
	hlgA	*hlgB*	*hlgC*	*lukD*	*lukE*	*sea*	*sed*	*sej*	*ser*	*seg*	*sei*	*sem*	*sen*	*seo*	*seu*	*tst*	*sep*	*lukF-PV*	*lukS-PV*	*sec*	*sec3*	*sel*	*aur*	*splA*	*splB*	*splE*	*sak*	*scn*
MRSA_01	A	B	B	A	C	A	C	B	B														A	B	B	A	A	A
MRSA_02	A	B	B	C	C	C	C	B	B														A	B	B	A	A	A
MRSA_03	A	B	B	A	C	A	C	B	B														A	B	B	A	A	A
MRSA_04	A	B	B	A	C	A																	A	B	B	A	A	A
MRSA_05	A	B	B	A	C	A	B	B	B														C	B	B	A	A	A
MRSA_06	A	B	B	A	C	C	B	B	B														B	B	B	A	A	A
MRSA_07	A	B	B	A	C	A	C	B	B														A	B	B	A	A	A
MRSA_08	A	B	B	A	C	A	B	B	B														C	B	B	A	A	A
MRSA_09	A	B	B	A	C	A																	C	B	B	A	A	A
MRSA_10	A	B	B	A	C	A	C	B	B														A	B	B	A	C	A
MRSA_11	A	B	B	C	C	C	B	B	B														C	B	B	A	B	A
MRSA_12	A	B	B	A	C	A	C	B	B														A	B	B	A	A	A
MRSA_13	B	A	A							B	B	B	A	A	A								A					
MRSA_14	B	A	A							B	B	B	A	A	A	A							A				A	A
MRSA_15	B	A	A							B	B	C	A	A	A	A							A				A	B
MRSA_16	B	A	A							B	B	B	A	A	A	A							A				B	A
MRSA_17	B	A	A							B	B	B	A	A	A	A							A				A	A
MRSA_18	B	A	A							B	B	B	A	A	A	A							A				A	A
MRSA_19	B	A	A							B	B	B	A	A	A	A							A				B	A
MRSA_20	B	A	A							B	B	B	A	A	A	A							A					
MRSA_21	B	A	A							B	B	B	A	A	A	A							A				A	A
MRSA_22	A	A	A	A	B					B	A	A	A	A	C	A	A						A	C	A		A	A
MRSA_23	A	A	A	A	B					B	A	A	A	A	C	A	A						A	A	A		A	A
MRSA_24	A	A	A	A	A					C	A	A	A	A	C		A						A	A	A		A	A
MRSA_25	A	A	A	A	A					B	A	A	A	A	C		A						A	A	A		A	A
MRSA_26	A	A	A	A	A					B	A	A	A	A	C		A						A	A	A		A	A
MRSA_27	A	A	A	A	A					B	A	A	A	A	C		A						A	A	A		A	A
MRSA_28	A	A	A	A	A					B	A	A	B	A	C		A						A	A	A		A	A
MRSA_29	A	A	A	A	B					B	A	A	A	A	C	A	A						A	A	A		A	A
MRSA_30	A	A	A	A	B					B	A	A	A	B	C	A	A						A	A	A		A	A
MRSA_31	A	A	A	B	A					C	A	A	A	A	C		A						A	A	A		A	A
MRSA_32	A	A	A	B	A		A	B	B	C	A	A	A	A	C		A						C	A	A		C	A
MRSA_33	A	A	B	A	A		A	B	B	C	A	A	A	B	C		A						A	A	A		A	A
MRSA_34	A	A	B	A	A					B	A	A	A	B	C		A						A	A	A		A	A
MRSA_35	A	A	B	A	A		B	B	B	B	B	A	A	A	C		A						A	A	A		A	A
MRSA_36	A	A	B	A	A		B		B	B	B	A	A	A	C		A						C	A	A		A	A
MRSA_37	A	A	B	A	A		B	B	B	B	B	A	A	A	C		A						A	A	A		A	A
MRSA_38	A	A	B	A	A		B	B	B	B	B	A	A	A	C		A						A	A	A		A	A
MRSA_39	A	A	C			A				B	B	B	A	A	B			A	A	A	A	C	B					A
MRSA_40	B	A	C			A				B	B	B	B	A	B			A	A	A	A	C	B					A
MRSA_41	A	C	C			A				B	B	B	A	A	B			A	A	A	A	C	B					A
MRSA_42	A	A	C			A				B	B	B	A	A	B			A	A	A	A	C	B					A

Category A (dark green): Gene Size in the Input Genome = Reference Gene Size. Identity = 100%

Category B (light green): Gene Size in the Input Genome = Reference Gene Size. Identity < 100%

Category C (gray): Gene Size in the Input Genome ≠ Reference Gene Size. Identity ≤ 100%

Table S19. Table of results from genomic analyses conducted using the VirulenceFinder service with MRSA genomes obtained through the ONT48hA protocol.

ISOLATE	GENE																											
	hlgA	*hlgB*	*hlgC*	*lukD*	*lukE*	*sea*	*sed*	*sej*	*ser*	*seg*	*sei*	*sem*	*sen*	*seo*	*seu*	*tst*	*sep*	*lukF-PV*	*lukS-PV*	*sec*	*sec3*	*sel*	*aur*	*splA*	*splB*	*splE*	*sak*	*scn*
MRSA_01	B	B	B	B	C	B	B	B	B														B	B	B	B	B	B
MRSA_02	B	B	B	B	C	B	B	B	B														B	B	B	B	B	B
MRSA_03	B	B	B	B	C	B	B	B	B														B	B	B	B	B	B
MRSA_04	B	B	B	B	C	B																	B	B	B	B	A	B
MRSA_05	B	B	B	B	C	B	B	B	B														B	B	B	B	B	B
MRSA_06	B	B	B	B	C	B	B	B	B														B	B	B	B	B	A
MRSA_07	B	B	B	B	C	B	B	B	B														A	B	B	B	B	B
MRSA_08	B	B	B	B	C	B	B	B	B														B	B	B	B	B	A
MRSA_09	B	B	B	B	C	B																	B	B	B	B	B	B
MRSA_10	B	B	B	B	C	B	B	B	B														B	B	B	B	B	B
MRSA_11	A	B	B	B	C	B	B	B	B														B	B	B	B	B	B
MRSA_12	B	B	B	B	C	B	B	B	B														A	B	B	B	B	B
MRSA_13	B	B	A							B	B	B	B	A	A	B							B					
MRSA_14	B	B	A							B	B	B	B	A	A	B							B				B	B
MRSA_15	B	B	A							B	B	B	B	A	A	B							B				B	B
MRSA_16	B	B	B							B	B	B	B	A	A	B							B				B	B
MRSA_17	B	B	B							B	B	B	B	A	A	B							B				B	B
MRSA_18	B	B	A							B	B	B	B	A	A	B							B				B	B
MRSA_19	B	B	A							B	B	B	B	A	A	B							A				B	B
MRSA_20	B	B	A							B	B	B	B	A	B	B							B					
MRSA_21	B	B	A							B	B	B	B	A	A	B							B				B	A
MRSA_22	B	B	A	B	B					B	B	A	B	A	B	B	B						A	A	A		B	B
MRSA_23	A	B	A	B	B					B	B	A	B	A	B	B	B						B	A	B		B	B
MRSA_24	B	B	A	B	B					B	B	B	B	A	B		B						B	B	B		B	B
MRSA_25	B	B	A	B	B					B	B	B	B	A	B		B						A	A	A		B	B
MRSA_26	B	B	B	B	B					B	B	B	B	A	C		B						B	A	A		B	A
MRSA_27	B	B	A	B	B					B	B	B	B	A	B		B						B	A	B		B	B
MRSA_28	A	B	A	B	B					B	B	B	B	A	B		B						B	A	B		B	B
MRSA_29	B	B	A	B	B					B	B	B	B	A	B	B	B						B	A	A		B	A
MRSA_30	B	B	B	B	B					B	B	B	B	B	B	B	B						B	A	B		B	B
MRSA_31	A	B	B	B	A		B	B	B	B	B	B	B	A	B		B						B	A	A		B	B
MRSA_32	B	B	A	B	B		B	B	B	B	B	B	B	A	B		B						A	A	A		B	B
MRSA_33	A	B	B	B	A		B	B	B	B	B	B	B	B	B		B						B	A	B		B	A
MRSA_34	B	B	B	B	A		B	B	B	B	B	B	B	B	B		B						B	A	A		B	B
MRSA_35	B	B	B	B	A		B	B	B	B	B	B	B	A	B		B						B	A	A		B	A
MRSA_36	B	B	B	B	A		B	B	B	B	B	B	B	A	B		B						A	A	A		B	B
MRSA_37	B	B	B	B	B		B	B	B	B	B	B	B	A	B		B						B	A	A		B	B
MRSA_38	B	B	B	B	B			B	B	B	B	A	B	A	B		B						B	A	A		B	B
MRSA_39	B	B	A			B				B	B	B	B	A	B			B	B	B	B	C	B					B
MRSA_40	B	B	A			B				B	B	B	B	A	B			B	B	B	B	C	B					B
MRSA_41	B	B	A			B				B	B	B	B	A	B			B	B	B	B	C	B					B
MRSA_42	B	B	A			B				B	B	B	B	A	B			B	B	B	B	C	B					B

Category A (dark green): Gene Size in the Input Genome = Reference Gene Size. Identity = 100%

Category B (light green): Gene Size in the Input Genome = Reference Gene Size. Identity < 100%

Category C (gray): Gene Size in the Input Genome ≠ Reference Gene Size. Identity ≤ 100%

Table S20. Table of results from genomic analyses conducted using the VirulenceFinder service with MRSA genomes obtained through the ONT48hB protocol.

ISOLATE	GENE																											
	hlgA	*hlgB*	*hlgC*	*lukD*	*lukE*	*sea*	*sed*	*sej*	*ser*	*seg*	*sei*	*sem*	*sen*	*seo*	*seu*	*tst*	*sep*	*lukF-PV*	*lukS-PV*	*sec*	*sec3*	*sel*	*aur*	*splA*	*splB*	*splE*	*sak*	*scn*
MRSA_01	A	B	B	A	C	A	C	B	B														A	B	B	A	A	A
MRSA_02	A	B	B	C	C	A	C	B	B														C	B	B	A	C	A
MRSA_03	A	B	B	A	C	A	C	B	B														A	B	B	A	A	A
MRSA_04	A	B	B	A	C	A																	C	B	B	A	A	A
MRSA_05	A	B	C	A	C	A	B	B	B														A	B	B	A	A	A
MRSA_06	A	B	B	A	C	A	C	B	B														C	B	B	A	C	A
MRSA_07	A	B	B	A	C	A	C	B	B														A	B	B	A	A	A
MRSA_08	A	B	B	A	C	A	B	B	B														A	B	B	A	A	A
MRSA_09	A	B	B	A	C	A																	A	B	B	A	C	A
MRSA_10	A	B	B	A	C	A	C	B	B														A	B	B	A	C	A
MRSA_11	A	B	B	C	C	C	C	B	B														C	B	B	A	A	A
MRSA_12	A	B	B	A	C	A	C	B	C														A	B	B	A	A	A
MRSA_13	B	A	A							B	B	B	A	A	A	A							A					
MRSA_14	B	A	A							B	B	B	A	A	A	A							A				A	A
MRSA_15	B	A	A							B	B	B	A	A	A	A							A				A	B
MRSA_16	B	A	A							B	B	B	A	A	A	A							A				B	A
MRSA_17	B	A	A							B	B	B	A	A	A	A							A				A	A
MRSA_18	B	A	A							B	B	B	A	A	A	A							A				A	A
MRSA_19	B	A	A							B	B	B	A	A	A	A							A				B	A
MRSA_20	B	A	A							B	B	B	A	A	A	A							A					
MRSA_21	B	A	A							B	B	B	A	A	A	A							A				A	A
MRSA_22	A	A	A	A	B					B	A	A	A	A	C	A	A						A	C	A		A	A
MRSA_23	A	A	A	A	B					B	A	A	A	A	C	A	A						A	A	A		A	A
MRSA_24	A	A	A	A	A					C	A	A	A	A	C		A						A	A	A		A	A
MRSA_25	A	A	A	A	A					B	A	A	A	A	C		A						A	A	A		A	A
MRSA_26	A	A	A	A	A					B	A	A	A	A	C		A						A	A	A		A	A
MRSA_27	A	A	A	A	A					B	A	A	A	A	C		A						A	A	A		A	A
MRSA_28	A	A	A	A	A					C	A	A	A	A	C		A						A	A	A		A	A
MRSA_29	A	A	A	A	B					B	A	A	A	A	C	A	A						A	A	A		A	A
MRSA_30	A	A	A	A	B					B	A	A	A	B	C	A	A						A	A	A		A	A
MRSA_31	A	A	A	B	A					C	A	A	A	A	C		A						A	A	A		A	A
MRSA_32	A	A	A	B	A		A	B	B	B	A	A	A	A	C		A						A	A	A		A	A
MRSA_33	A	A	B	A	A		A	B	B	C	A	A	A	B	C		A						A	A	A		A	A
MRSA_34	A	A	B	A	A					B	A	A	A	B	C		A						A	A	A		A	A
MRSA_35	A	A	B	A	A		B	B	B	B	B	A	A	A	C		A						A	A	A		A	A
MRSA_36	A	A	B	A	A		B	B	B	B	B	A	A	A	C		A						C	A	A		A	A
MRSA_37	A	A	B	A	A					B	B	A	A	A	C		A						A	A	A		C	A
MRSA_38	A	A	B	A	A		B	B	B	B	B	A	A	A	C		A						A	A	A		A	A
MRSA_39	A	A	C			A				B	B	B	A	A	B			A	A	A	A	C	B					A
MRSA_40	A	A	C			A				B	B	B	B	A	B			A	A	A	A	C	B					A
MRSA_41	A	A	C			A				B	B	B	A	A	B			A	A	A	A	C	B					A
MRSA_42	A	A	C			A				B	B	B	A	A	B			A	A	A	A	C	B					A

Category A (dark green): Gene Size in the Input Genome = Reference Gene Size. Identity = 100%

Category B (light green): Gene Size in the Input Genome = Reference Gene Size. Identity < 100%

Category C (gray): Gene Size in the Input Genome ≠ Reference Gene Size. Identity ≤ 100%

Table S21. Table of results from genomic analyses conducted using the VirulenceFinder service with MRSA genomes obtained through the IT protocol.

ISOLATE	GENE																										
	hlgA	*hlgB*	*hlgC*	*lukD*	*lukE*	*sea*	*sed*	*sej*	*ser*	*seg*	*sei*	*sem*	*sen*	*seo*	*seu*	*tst*	*sep*	*lukF-PV*	*lukS-PV*	*sec*	*sec3*	*sel*	*aur*	*splA*	*splB*	*splE*	*sak*	*scn*
MRSA_01	A	B	B	A	C	A	C	C	B														A	B	B	A	A	A
MRSA_02	A	B	B	A	C	A	C	B	B														A	B	B	A	A	A
MRSA_03	A	B	B	A	C	A	C	B	B														A	B	B	A	A	A
MRSA_04	A	B	B	A	C	A																	A	B	B	A	A	A
MRSA_05	A	B	B	A	C	A	C	B	B														A	B	B	A	A	A
MRSA_06	A	B	B	A	C	A	C	B	B														A	B	B	A	A	A
MRSA_07	A	B	B	A	C	A	C	B	C														A	B	B	A	A	A
MRSA_08	A	B	B	C	C	A	C	B	C														A	B	B	A	A	A
MRSA_09	A	B	B	A	C	A																	A	B	B	A	A	A
MRSA_10	A	B	B	A	C	A	C	B	B														A	B	B	A	A	A
MRSA_11	A	B	B	A	C	A	C	B	B														A	B	B	A	A	A
MRSA_12	A	B	B	A	C	A	C	B	B														A	B	B	A	A	A
MRSA_13	B	A	A							B	B	B	A	A	A	A							A					
MRSA_14	B	A	A							B	B	B	A	A	A	A							A				A	A
MRSA_15	B	A	A							B	B	B	A	A	A	A							A				A	B
MRSA_16	B	A	A							B	B	B	A	A	A	A							A				B	A
MRSA_17	B	A	A							B	B	B	A	A	A	A							A				A	A
MRSA_18	B	A	A							B	B	B	A	A	A	A							A				A	A
MRSA_19	B	A	A							B	B	B	A	A	A	A							A				B	A
MRSA_20	B	A	A							B	B	B	A	A	A	A							A					
MRSA_21	B	A	A							B	B	B	A	A	A	A							A				A	A
MRSA_22	A	A	A	A	B					B	A	A	A	A	C		A						A	A	A		A	A
MRSA_23	A	A	A	A	B					B	A	A	A	A	C		A						A	A	A		A	A
MRSA_24	A	A	A	A	A					C	A	A	A	A	C		A						A	A	A		A	A
MRSA_25	A	A	A	A	A					B	A	A	A	A	C		A						A	A	A		A	A
MRSA_26	A	A	A	A	A					B	A	A	A	A	C		A						A	A	A		A	A
MRSA_27	A	A	A	A	A					B	A	A	A	A	C		A						A	A	A		A	A
MRSA_28	A	A	A	A	A					B	A	A	A	A	C		A						A	A	A		A	A
MRSA_29	A	A	A	A	B					B	A	C	A	A	C	C	A						A	A	A		A	A
MRSA_30	A	A	A	A	B					B	A	A	A	B	C	A	A						A	A	A		A	A
MRSA_31	A	A	A	B	A		A	B	B	B	A	A	A	A	C		A						A	A	A		A	A
MRSA_32	A	A	A	B	A		A	C	B	B	A	A	A	A	C		A						A	A	A		A	A
MRSA_33	A	A	B	A	A		A	B	B	B	A	A	A	B	C		A						A	A	A		A	A
MRSA_34	A	A	B	A	A		A	B	B	B	A	A	A	B	C		A						A	A	A		A	A
MRSA_35	A	A	B	A	A		B	B	B	B	B	A	A	A	C		A						A	A	A		A	A
MRSA_36	A	A	B	A	A		B	B	B	B	B	A	A	A	C		A						A	A	A		A	A
MRSA_37	A	A	B	A	A		B	B	B	B	B	A	A	A	C		A						A	A	A		A	A
MRSA_38	A	A	B	A	A		B	B	B	B	B	A	A	C	C		A						A	A	A		A	A
MRSA_39	A	A	A			A				B	B	B	A	A	B			A	A	A	A	C	B					A
MRSA_40	A	A	A			A				C	C	B	B	A	B			A	A	A	A	C	B					A
MRSA_41	A	A	A			A				B	C	B	A	A	B			A	A	A	A	C	B					A
MRSA_42	A	A	A			A				B	B	B	A	A	B			A	A	A	A	C	C					A

Category A (dark green): Gene Size in the Input Genome = Reference Gene Size. Identity = 100%

Category B (light green): Gene Size in the Input Genome = Reference Gene Size. Identity < 100%

Category C (gray): Gene Size in the Input Genome ≠ Reference Gene Size. Identity ≤ 100%

Table S22. Table of results from genomic analyses conducted using the VirulenceFinder service with MRSA genomes obtained through the Hybrid protocol.

ISOLATE	GENE																											
	hlgA	*hlgB*	*hlgC*	*lukD*	*lukE*	*sea*	*sed*	*sej*	*ser*	*seg*	*sei*	*sem*	*sen*	*seo*	*seu*	*tst*	*sep*	*lukF-PV*	*lukS-PV*	*sec*	*sec3*	*sel*	*aur*	*splA*	*splB*	*splE*	*sak*	*scn*
MRSA_01	A	B	B	A	C	A	C	B	B														A	B	B	A	A	A
MRSA_02	A	B	B	A	C	A	C	B	B														A	B	B	A	A	A
MRSA_03	A	B	B	A	C	A	C	B	B														A	B	B	A	A	A
MRSA_04	A	B	B	A	C	A																	A	B	B	A	A	A
MRSA_05	A	B	B	A	C	A	C	B	B														A	B	B	A	A	A
MRSA_06	A	B	B	A	C	A	C	B	B														A	B	B	A	A	A
MRSA_07	A	B	B	A	C	A	C	B	B														A	B	B	A	A	A
MRSA_08	A	B	B	A	C	A	C	B	B														A	B	B	A	A	A
MRSA_09	A	B	B	A	C	A																	A	B	B	A	A	A
MRSA_10	A	B	B	A	C	A	C	B	B														A	B	B	A	A	A
MRSA_11	A	B	B	A	C	A	C	B	B														A	B	B	A	A	A
MRSA_12	A	B	B	A	C	A	C	B	B														A	B	B	A	A	A
MRSA_13	B	A	A							B	B	B	A	A	A	A							A					
MRSA_14	B	A	A							B	C	B	A	A	A	A							A				A	A
MRSA_15	B	A	A							B	B	B	A	A	A	A							A				A	B
MRSA_16	B	A	A							B	B	B	A	A	A	A							A				B	A
MRSA_17	B	A	A							B	B	B	A	A	A	A							A				A	A
MRSA_18	B	A	A							B	B	B	A	A	A	A							A				A	A
MRSA_19	B	A	A							B	B	B	A	A	A	A							A				B	A
MRSA_20	B	A	A							B	B	B	A	A	A	A							A					
MRSA_21	B	A	A							B	B	B	A	A	A	A							A				A	A
MRSA_22	A	A	A	A	B					B	A	A	A	A	C	A	A						A	A	A		A	A
MRSA_23	A	A	A	A	B					B	A	A	A	A	C	A	A						A	A	A		A	A
MRSA_24	A	A	A	A	A					B	A	A	A	A	C		A						A	A	A		A	A
MRSA_25	A	A	A	A	A					B	A	A	A	A	C		A						A	A	A		A	A
MRSA_26	A	A	A	A	A					B	A	A	A	A	C		A						A	A	A		A	A
MRSA_27	A	A	A	A	A					B	A	A	A	A	C		A						A	A	A		A	A
MRSA_28	A	A	A	A	A					B	A	A	A	A	C		A						A	A	A		A	A
MRSA_29	A	A	A	A	B					B	A	A	A	A	C	A	A						A	A	A		A	A
MRSA_30	A	A	A	A	B					B	A	A	A	B	C	A	A						A	A	A		A	A
MRSA_31	A	A	A	B	A		A	B	B	B	A	A	A	A	C		A						A	A	A		A	A
MRSA_32	A	A	A	B	A		A	B	B	B	A	A	A	A	C		A						A	A	A		A	A
MRSA_33	A	A	B	A	A		A	B	B	B	A	A	A	B	C		A						A	A	A		A	A
MRSA_34	A	A	B	A	A		A	B	B	B	A	A	A	B	C		A						A	A	A		A	A
MRSA_35	A	A	B	A	A		B	B	B	B	B	A	A	A	C		A						A	A	A		C	A
MRSA_36	A	A	B	A	A		B	B	B	B	B	A	A	A	C		A						A	A	A		A	A
MRSA_37	A	A	B	A	A		B	B	B	B	B	A	A	A	C		A						A	A	A		A	A
MRSA_38	A	A	B	A	A		B	B	B	B	B	A	A	A	C		A						A	A	A		C	A
MRSA_39	A	A	A			A				B	B	B	A	A	B			A	A	A	A	C	B					A
MRSA_40	A	A	A			A				B	B	B	B	B	B			A	A	A	A	C	B					A
MRSA_41	A	A	A			A				B	B	B	B	A	B			A	A	A	A	C	B					A
MRSA_42	A	A	A			A				B	C	C	B	A	B			A	A	A	A	C	B					A

Category A (dark green): Gene Size in the Input Genome = Reference Gene Size. Identity = 100%

Category B (light green): Gene Size in the Input Genome = Reference Gene Size. Identity < 100%

Category C (gray): Gene Size in the Input Genome ≠ Reference Gene Size. Identity ≤ 100%

Table S23. Table of results from genomic analyses conducted using the PlasmidFinder and MobileElementFinder services with MRSA genomes obtained through the ONT20h protocol.

ISOLATE	MOBILE GENE ELEMENT															
	rep20	rep7c	repUS43	rep5a	rep16	repUS5	rep7a	rep21	Tn6009	ISSau3	ISSau5	ISSau2	ISSep3	ISSau6	IS256	ISSau8
MRSA_01	A	B	A						B	C	C					
MRSA_02	A	B	A						B	C	C					
MRSA_03	A	B	A						B	C	C					
MRSA_04		B	A						B	C	C					
MRSA_05	A	B	A						B	C	B					
MRSA_06	A	B	A						B	C	B					
MRSA_07	A	B	A						B	C	C					
MRSA_08	A	B	A						B	C	C					
MRSA_09		B	A						B	C	B					
MRSA_10	A	B	A						B	C	C					
MRSA_11	A	B	A						B	C	B					
MRSA_12	A	B	A						B	C	C					
MRSA_13	A			C						C	C	B	C	B		
MRSA_14	A			A						C	C	B	C	B		
MRSA_15	A			A						C	C	B	C	B		
MRSA_16											C	B	C			
MRSA_17					A	B					C	B	C			
MRSA_18					A	B					C	B	C			
MRSA_19	A			A			A			C	C	B	C	B		
MRSA_20											C	B	C			
MRSA_21											C	B	C			
MRSA_22	A													C		
MRSA_23	A													C		
MRSA_24																
MRSA_25	A													C		
MRSA_26														C		
MRSA_27											C					
MRSA_28											C					
MRSA_29	A													C		
MRSA_30	A													C		
MRSA_31															B	
MRSA_32	A														B	
MRSA_33	A															
MRSA_34																
MRSA_35	A										C					
MRSA_36	A										C					
MRSA_37	A															
MRSA_38	A										C					
MRSA_39										C					B	C
MRSA_40										C					B	C
MRSA_41										C					B	C
MRSA_42								A		C					B	C

A — Category A (dark green): Gene Size in the Input Genome = Reference Gene Size. Identity = 100%
B — Category B (light green): Gene Size in the Input Genome = Reference Gene Size. Identity < 100%
C — Category C (gray): Gene Size in the Input Genome ≠ Reference Gene Size. Identity ≤ 100%

Table S24. Table of results from genomic analyses conducted using the PlasmidFinder and MobileElementFinder services with MRSA genomes obtained through the ONT48hA protocol.

ISOLATE	MOBILE GENE ELEMENT																	
	rep20	rep7c	repUS43	rep5a	rep16	repUS5	rep7a	rep21	repUS19	repUS21	Tn6009	ISSau3	ISSau5	ISSau2	ISSep3	ISSau6	IS256	ISSau8
MRSA_01	B	B	A						B		B	C	C					
MRSA_02	B	B	C						B		C	C	C					
MRSA_03	B	B	A								B	C	B					
MRSA_04		B	A						B		B	C	C					
MRSA_05	B	B	B						B		C	C	C					
MRSA_06	B	B	B						B		C	C	C					
MRSA_07	B	B	A						B		B	C	C		C			
MRSA_08	B	B	B						B		C	C	C					
MRSA_09		B	B						B		C	C	C					
MRSA_10	A	B	A								B	C	C					
MRSA_11	B	B	B						B		C	C	C					
MRSA_12	B	B	A								C	C	B					
MRSA_13	B			B								C	C	C		B		
MRSA_14	A			B								C	C	C		B		
MRSA_15													C	C	C			
MRSA_16	B						A						C	C	C			
MRSA_17					B	B			B				C	C				
MRSA_18					B	B							C	C				
MRSA_19	B			A			A					C	C	C		B		
MRSA_20													C	C				
MRSA_21													C	C				
MRSA_22	B															C		
MRSA_23	B															C		
MRSA_24										B								
MRSA_25	B									B						C		
MRSA_26	B									B						B		
MRSA_27										B			C					
MRSA_28										B			C					
MRSA_29	B									B						C		
MRSA_30	A									B						C		
MRSA_31	B									B							C	
MRSA_32	B									B							C	
MRSA_33	B																	
MRSA_34	B									B								
MRSA_35	B						B						C					
MRSA_36	B						A						C					
MRSA_37	A						A											
MRSA_38	A						A						C					
MRSA_39								B	C			C					C	C
MRSA_40								B	B			C					C	C
MRSA_41								B	B			C					C	C
MRSA_42								B		B		C					C	C

A — Category A (dark green): Gene Size in the Input Genome = Reference Gene Size. Identity = 100%

B — Category B (light green): Gene Size in the Input Genome = Reference Gene Size. Identity < 100%

C — Category C (gray): Gene Size in the Input Genome ≠ Reference Gene Size. Identity ≤ 100%

Table S25. Table of results from genomic analyses conducted using the PlasmidFinder and MobileElementFinder services with MRSA genomes obtained through the ONT48hB protocol.

ISOLATE	MOBILE GENE ELEMENT													
	rep20	rep7c	repUS43	rep5a	rep16	repUS5	Tn6009	ISSau3	ISSau5	ISSau2	ISSep3	ISSau6	IS256	ISSau8
MRSA_01	A	B	A				B	C	C					
MRSA_02	A	B	A				B	C	B					
MRSA_03	A	B	A				B	C	C					
MRSA_04		B	A				B	C	C					
MRSA_05	A	B	A				B	C	C					
MRSA_06	A	B	A				B	C	C					
MRSA_07	A	B	A				B	C	C					
MRSA_08	C	B	A				B	C	B					
MRSA_09		B	A				B	C	C					
MRSA_10	A	B	A				B	C	C					
MRSA_11	A	B	A				B	C	C					
MRSA_12	A	B	A				C	C	C					
MRSA_13	A			A				C	C	B	C	B		
MRSA_14	A			A				C	C	B	C	B		
MRSA_15	A			A				C	C	B	C	B		
MRSA_16									C	B	C			
MRSA_17					A	B			C	B	C			
MRSA_18					A	B			C	B	C			
MRSA_19	A			A				C	C	B	C	B		
MRSA_20									C	B	C			
MRSA_21									C	B	C			
MRSA_22	A											C		
MRSA_23	A											C		
MRSA_24														
MRSA_25	A											C		
MRSA_26												C		
MRSA_27									C					
MRSA_28									B					
MRSA_29												C		
MRSA_30												C		
MRSA_31													B	
MRSA_32	A												B	
MRSA_33	A													
MRSA_34														
MRSA_35	A								C					
MRSA_36	A								B					
MRSA_37														
MRSA_38	A								C					
MRSA_39								C					B	C
MRSA_40								C					B	C
MRSA_41								C					B	C
MRSA_42								C					B	C

A Category A (dark green): Gene Size in the Input Genome = Reference Gene Size. Identity = 100%
B Category B (light green): Gene Size in the Input Genome = Reference Gene Size. Identity < 100%
C Category C (gray): Gene Size in the Input Genome ≠ Reference Gene Size. Identity ≤ 100%

Table S26. Table of results from genomic analyses conducted using the PlasmidFinder and MobileElementFinder services with MRSA genomes obtained through the IT protocol.

ISOLATE	MOBILE GENE ELEMENT																
	rep20	rep7c	repUS43	rep5a	rep16	repUS5	rep7a	rep21	repUS19	repUS21	Tn6009	ISSau3	ISSau5	ISSau2	ISSau6	IS256	ISSau8
MRSA_01	A	B	A						A		B		B				
MRSA_02	A	B	A						A		B		B				
MRSA_03	A	B	A						A		B		B				
MRSA_04		B	A						C		B		B				
MRSA_05	A	B	A										B				
MRSA_06	A	B	A						A		B		B				
MRSA_07	A	B	A						C		B	C	B				
MRSA_08	A	B	A								B		B				
MRSA_09		B	A						A		B		B				
MRSA_10	A	B	A								B		B				
MRSA_11	A	B	A								B		B				
MRSA_12	A	B	A								B		B				
MRSA_13	A			A									B	B	B		
MRSA_14	A			A									B	B	B		
MRSA_15	A			A								C	B	B	B		
MRSA_16													B	B			
MRSA_17					A	B							B	B			
MRSA_18					A	B							B	B			
MRSA_19	A			A			C						B	B	B		
MRSA_20													B	B			
MRSA_21													B	B			
MRSA_22	A														C		
MRSA_23	A														C		
MRSA_24										C							
MRSA_25	A									C					C		
MRSA_26	A									C					C		
MRSA_27													B				
MRSA_28													B				
MRSA_29	A									A					C		
MRSA_30	A									C					C		
MRSA_31	A															B	
MRSA_32	A															B	
MRSA_33	A																
MRSA_34	A																
MRSA_35	A																
MRSA_36	A												B				
MRSA_37	A																
MRSA_38	A												B				
MRSA_39								A								B	C
MRSA_40								C									C
MRSA_41								C									C
MRSA_42								C								B	C

A — Category A (dark green): Gene Size in the Input Genome = Reference Gene Size. Identity = 100%

B — Category B (light green): Gene Size in the Input Genome = Reference Gene Size. Identity < 100%

C — Category C (gray): Gene Size in the Input Genome ≠ Reference Gene Size. Identity ≤ 100%

Table S27. Table of results from genomic analyses conducted using the PlasmidFinder and MobileElementFinder services with MRSA genomes obtained through the Hybrid protocol.

ISOLATE	MOBILE GENE ELEMENT																	
	rep20	rep7c	repUS43	rep5a	rep16	repUS5	rep7a	rep21	repUS19	repUS21	Tn6009	ISSau3	ISSau5	ISSau2	ISSep3	ISSau6	IS256	ISSau8
MRSA_01	A	B	A						A		B	C	B					
MRSA_02	A	B	A						C		B	C	B					
MRSA_03	A	B	A						A		B	C	B					
MRSA_04		B	A						A		B	C	B					
MRSA_05	A	B	A								B	C	B					
MRSA_06	A	B	A						A		B	C	B					
MRSA_07	A	B	A						A		B	C	B					
MRSA_08	A	B	A								B	C	B					
MRSA_09		B	A						C		B	C	B					
MRSA_10	A	B	A								B	C	B					
MRSA_11	A	B	A								B	C	B					
MRSA_12	A	B	A								B	C	B					
MRSA_13	A			A								C	B	B	C	B		
MRSA_14	A			A								C	B	B	C	B		
MRSA_15	A			A								C	B	B	C	B		
MRSA_16													B	B	C			
MRSA_17					A	B							B	B	C			
MRSA_18					A	B							B	B	C			
MRSA_19	A			A			A					C	B	B		B		
MRSA_20													B	B	C			
MRSA_21													B	B	C			
MRSA_22	C															C		
MRSA_23	C															C		
MRSA_24										A								
MRSA_25	C									C						C		
MRSA_26	C									C						C		
MRSA_27													B					
MRSA_28													B					
MRSA_29	C															C		
MRSA_30	C															C		
MRSA_31	A																B	
MRSA_32	A																B	
MRSA_33	A																	
MRSA_34	A																	
MRSA_35	A												B					
MRSA_36	A												B					
MRSA_37	A																	
MRSA_38	A												B					
MRSA_39								C				C					B	C
MRSA_40								C				C					B	C
MRSA_41								C				C					C	C
MRSA_42												C					B	C

A — Category A (dark green): Gene Size in the Input Genome = Reference Gene Size. Identity = 100%

B — Category B (light green): Gene Size in the Input Genome = Reference Gene Size. Identity < 100%

C — Category C (gray): Gene Size in the Input Genome ≠ Reference Gene Size. Identity ≤ 100%

Table S28. Table of results from genomic analyses conducted using the PlasmidFinder and MobileElementFinder services with ESBL-Kp genomes obtained through the ONT20h protocol.

ISOLATE	MOBILE GENE ELEMENT																						
	IncFIB(K)	IncFII(K)	Col440I	IncFIA(pBK30683)	IS6100	ISKpn14	ISKpn33	ISEcl1	ISKpn26	IS102	IS903	ISKpn142	ISKpn263	ISEc33	ISSen4	ISEch12	Tn5403	IS26	IS5075	IS5	ISEc52	ISSty2	ISKpn262
Kp_01	B	B			A	B	A	B	B	B	B	B	B	B	C	C							
Kp_02	B	B			A	C	C	C	C	C	C			C	C	C							C
Kp_03			B	B	A					C						C	C	A	B	B	C	C	
Kp_04			B	B						C						C	C	A	B	B	C	C	
Kp_05			B	B	A					C						C	C	A	B	B	C	C	
Kp_06			B	B	A					C						C	C	A	B	B	C	C	

Category A (dark green): Gene Size in the Input Genome = Reference Gene Size. Identity = 100%

Category B (light green): Gene Size in the Input Genome = Reference Gene Size. Identity < 100%

Category C (gray): Gene Size in the Input Genome ≠ Reference Gene Size. Identity ≤ 100%

Table S29. Table of results from genomic analyses conducted using the PlasmidFinder and MobileElementFinder services with ESBL-Kp genomes obtained through the ONT48hB protocol.

ISOLATE	MOBILE GENE ELEMENT																					
	IncFIB(K)	IncFII(K)	IncFIA(pBK30683)	IS6100	ISKpn14	ISKpn33	ISEcl1	ISKpn26	IS102	IS903	ISEc33	ISSen4	ISEch12	Tn5403	IS26	IS5075	IS5	IS262	ISSty2	IS61004	ISEc52	ISEc9
Kp_01	B	B		A	B	C	B	B	B	B	B	C	C									
Kp_02	B	B		A	B	C	B	B	B	C	B	C	C		A							C
Kp_03			B						C				C	C	A	B	B	A	C			
Kp_04			B						C				C	C	B	B	B	A	C	A	C	
Kp_05			B	A					C				C	C	A	B	B		C		C	
Kp_06			B	A					C				C	C	A	B	B		C		C	

Category A (dark green): Gene Size in the Input Genome = Reference Gene Size. Identity = 100%

Category B (light green): Gene Size in the Input Genome = Reference Gene Size. Identity < 100%

Category C (gray): Gene Size in the Input Genome ≠ Reference Gene Size. Identity ≤ 100%

Table S30. Table of results from genomic analyses conducted using the PlasmidFinder and MobileElementFinder services with ESBL-Kp genomes obtained through the IT protocol.

**ISOLATE**	**MOBILE GENE ELEMENT**		
	ISEch12	ISEc52	IS5
Kp_01	C		
Kp_02	C		
Kp_03	C	C	
Kp_04	C		B
Kp_05	C		B
Kp_06	C		B

A	Category A (dark green): Gene Size in the Input Genome = Reference Gene Size. Identity = 100%
B	Category B (light green): Gene Size in the Input Genome = Reference Gene Size. Identity < 100%
C	Category C (gray): Gene Size in the Input Genome ≠ Reference Gene Size. Identity ≤ 100%

Table S31. Results for Phenotypic Antimicrobial Susceptibility Testing (AST) Conducted on Clinical Isolates of MRSA. S = Susceptible (standard dosing); I = Susceptible (increased exposure); R = Resistant. Values in parentheses represent inhibition zone diameters in millimeters. AST are not available for the isolates in red.

**ISOLATE**	**Antimicrobian**																	
	**Ampicillin**	**Mupirocin**	**Cefoxitin**	**Ciprofloxacin**	**Erythromycin**	**fusidic acid**	**Gentamicin**	**Clindamicin**	**Chloranfenicol**	**Linezolide**	**Oxacillin**	**Penicilin G**	**Rifampicin**	**Teicoplanine**	**Tetracycline**	**Trimethoprim sulfamethoxazo le**	**Vanconicin**	**Tigecicline**
**MRSA_01**	R	S (34)	R (6)	I (23)	S (28)	S (30)	S (25)	S (28)	S (24)	S (27)	R (6)	R (6)	S (33)	S (0.5)	R (6)	S (27)	S (1.0)	S (24)
**MRSA_02**	R	S (36)	R (11)	I (23)	S (28)	S (32)	S (25)	S (28)	S (23)	S (25)	R (6)	R (6)	S (34)	S (0.5)	R (8)	S (31)	S (1.0)	S (25)
**MRSA_03**	R	S(34)	R (11)	I (22)	S (28)	S (32)	S (24)	S (27)	S (24)	S (34)	R (6)	R (6)	S (34)	S (0.5)	R (8)	S (29)	S (1.0)	S (24)
**MRSA_04**	R	S(38)	R(21)	I (24)	S (27)	S (33)	S (23)	S (28)	S (24)	S (24)	R (16)	R (23)	S (33)	S (0.5)	R (13)	S (23)	S (0.5)	S (25)
**MRSA_05**	R	S(40)	R(21)	I (24)	S (26)	S (34)	S (23)	S (24)	S (26)	S (28)	R (14)	R (6)	S (34)	S (0.5)	R (13)	S (21)	S (0.5)	S (25)
**MRSA_06**	R	S(38)	R(21)	I (23)	S (28)	S (34)	S (24)	S (24)	S (26)	S (28)	R (16)	R (6)	S (34)	S (0.5)	R (13)	S (22)	S (0.5)	S (27)
**MRSA_07**	R	S(39)	R(21)	I (25)	S (27)	S (33)	S (23)	S (25)	S (26)	S (26)	R (17)	R (6)	S (36)	S (0.5)	R (13)	S (25)	S (0.5)	S (26)
**MRSA_08**	R	S(39)	R(21)	I (26)	S (28)	S (36)	S (24)	S (26)	S (24)	S (27)	R (16)	R (6)	S (36)	S (0.5)	R (13)	S (24)	S (0.5)	S (28)
**MRSA_09**	R	S(40)	R(21)	I (26)	S (27)	S (34)	S (24)	S (25)	S (25)	S (26)	R (17)	R (22)	S (35)	S (0.5)	R (13)	S (24)	S (0.5)	S (28)
**MRSA_10**	R	S(39)	R(21)	I (25)	S (28)	S (34)	S (22)	S (26)	S (24)	S (27)	R (16)	R (6)	S (35)	S (0.5)	R (13)	S (24)	S (0.5)	S (27)
**MRSA_11**	R	S(37)	R(21)	I (24)	S (26)	S (34)	S (22)	S (25)	S (24)	S (26)	R (16)	R (6)	S (33)	S (0.5)	R (19)	S (24)	S (0.5)	S (27)
**MRSA_12**																		
**MRSA_13**	R	S (35)	R (13)	I (25)	S (26)	S (32)	S (24)	S (27)	S (26)	S (25)	R(16)	R (6)	S (32)	S (1.0)	S (29)	S (30)	S (1.0)	S (24)
**MRSA_14**	R	S (35)	R (14)	I (23)	S (27)	S (30)	S (25)	S (25)	S (24)	S (24)	R(14)	R (6)	S (32)	S (0.5)	S (27)	S (29)	S (0.5)	S (25)
**MRSA_15**	R	S (41)	R (21)	I (27)	S (26)	S (35)	S (24)	S (25)	S (24)	S (26)	R(15)	R (9)	S (36)	S (0.5)	S (31)	S (27)	S (0.5)	S (26)
**MRSA_16**	R	S (34)	R (13)	I (25)	S (27)	S (30)	S (24)	S (26)	S (24)	S (25)	R (10)	R (6)	S (28)	S (1.0)	S (32)	S (26)	S (0.5)	S (22)
**MRSA_17**	R	S (35)	R (14)	I (28)	S (29)	S (30)	S (26)	S (25)	S (23)	S (25)	R (6)	R (6)	S (32)	S (1.0)	S (28)	S (32)	S (0.5)	S (23)
**MRSA_18**	R	S (35)	R (14)	I (26)	S (28)	S (28)	S (25)	S (25)	S (22)	S (24)	R (6)	R (6)	S (32)	S (0.5)	S (27)	S (30)	S (0.5)	S (23)
**MRSA_19**	R	S (35)	R (13)	I (27)	S (28)	S (30)	S (25)	S (27)	S (23)	S (24)	R (6)	R (6)	S (36)	S (1.0)	R (10)	S (20)	S (0.5)	S (24)
**MRSA_20**																		
**MRSA_21**	R	S (34)	R (14)	I (28)	S(27)	S (29)	S (24)	S (24)	S (22)	S (24)	R (6)	R (6)	S (28)	S (1.0)	S (23)	S (30)	S (0.5)	S (23)
**MRSA_22**	R	S (35)	R (14)	I (27)	S (27)	S (28)	S (23)	S (25)	S (23)	S (24)	R (14)	R (15)	S (32)	S (1.0)	S (26)	S (29)	S (1.0)	S (23)
**MRSA_23**	R	S (33)	R (13)	I (25)	S (28)	S (30)	S (24)	S (27)	S (23)	S (25)	R (6)	R (6)	S (32)	S (1.0)	S (27)	S (31)	S (1.0)	S (24)
**MRSA_24**	R	S (34)	R (12)	I (25)	S (27)	S (30)	S (24)	S (26)	S (23)	S (25)	R (6)	R (6)	S (31)	S (1.0)	S (26)	S (28)	S (1.0)	S (23)
**MRSA_25**	R	S (35)	R (12)	I (26)	S (28)	S (28)	S (24)	S (27)	S (23)	S (23)	R (6)	R (6)	S (35)	S (0.5)	S (28)	S (28)	S (1.0)	S (24)
**MRSA_26**	R	S (37)	R (14)	I (27)	S (28)	S (32)	S (27)	S (29)	S (25)	S (27)	R (15)	R (6)	S (34)	S (0.5)	S (30)	S (30)	S (1.0)	S (24)
**MRSA_27**	R	S (35)	R (13)	I (23)	S (28)	R (23)	S (24)	S (26)	S (23)	S (24)	R (13)	R(18)	S (32)	S (1.0)	S (28)	S (30)	S (1.0)	S (23)
**MRSA_28**	R	S (33)	R (14)	I (22)	S (28)	R (22)	S (23)	S (27)	S (22)	S (24)	R (11)	R (22)	S (28)	S (0.5)	S (26)	S (30)	S (1.0)	S (23)
**MRSA_29**	R	S (35)	R (13)	I (25)	S (28)	S (28)	S (24)	S (28)	S (23)	S (24)	R (13)	R (6)	S (33)	S (0.5)	S (25)	S (31)	S (1.0)	S (23)
**MRSA_30**	R	S (35)	R (13)	I(25)	S (28)	S (30)	S (24)	S (28)	S (24)	S (28)	R (13)	R (6)	S (34)	S (1.0)	S (27)	S (32)	S (0.5)	S (24)
**MRSA_31**	R	S (35)	R (16)	I (25)	S (30)	S (28)	R (9)	S (28)	S (24)	S (24)	R (13)	R (12)	S (31)	S (0.5)	S (28)	S (32)	S (1.0)	S (24)
**MRSA_32**	R	S (35)	R (18)	I (28)	S (26)	S (28)	R (10)	S (26)	S (24)	S (24)	R (12)	R (11)	S (30)	S (1.0)	S (27)	S (31)	S (1.0)	S (24)
**MRSA_33**																		
**MRSA_34**																		
**MRSA_35**	R	S (35)	R (13)	R (6)	S (27)	S (28)	S (24)	S (27)	S (24)	S (26)	R (6)	R (6)	S (30)	S (0.5)	S (27)	S (30)	S (1.0)	S (24)
**MRSA_36**	R	S (35)	R (13)	R (6)	S (26)	S (32)	S (25)	S (27)	S (25)	S (25)	R (6)	R (6)	S (32)	S (0.5)	S (26)	S (29)	S (0.5)	S (24)
**MRSA_37**	R	S (34)	R (13)	R (6)	S (28)	S (30)	S (23)	S (26)	S (22)	S (23)	R (6)	R (6)	S (30)	S (1.0)	S (25)	S (30)	S (1.0)	S (22)
**MRSA_38**	R	S (34)	R (12)	R (6)	S (28)	S (30)	S (23)	S (27)	S (23)	S (25)	R (6)	R (6)	S (30)	S (1.0)	S (26)	S (29)	S (1.0)	S (22)
**MRSA_39**	R	S (34)	R (16)	R (16)	R (17)	S (30)	R (9)	S (27)	S (23)	S (24)	R (6)	R (6)	S (31)	S (1.0)	S (26)	S (22)	S (1.0)	S (23)
**MRSA_40**	R	S (33)	R (13)	R (13)	R (18)	S (30)	R (9)	S (27)	S (24)	S (24)	R (6)	R (6)	S (30)	S (1.0)	S (26)	S (20)	S (1.0)	S (23)
**MRSA_41**																		
**MRSA_42**																		

R	R - Resistant
I	I – Susceptible increasing exposure
S	S - Susceptible

Table S32. Results for Phenotypic Antimicrobial Susceptibility Testing Conducted on Clinical Isolates of ESBL-Kp. S = Susceptible (standard dosing); I = Susceptible (increased exposure); R = Resistant. Values in parentheses represent inhibition zone diameters in millimeters.

ISOLATE	Antimicrobian											
	**Amoxicillin-clavulanate**	**Ampicillin**	**Aztreonam**	**Cefotaxime**	**Ceftazidime**	**Cefuroxime**	**Piperacillin-tazobactam**	**Ciprofloxacin**	**Gentamicin**	**Meropenem**	**Trimethoprim sulfamethoxazol**	**Cefepime**
Kp_01	R	R	R	R	R	R	S	R	S	S	R	R
Kp_02	R	R	R	R	R	R	S	R	S	S	R	R
Kp_03	R	R	R	R	R	R	S	I	S	S	R	R
Kp_04	R	R	R	R	R	R	I	R	S	S	R	R
Kp_05	R	R	R	R	R	R	S	I	S	S	R	R
Kp_06	R	R	R	R	R	R	S	R	S	S	R	R

R	R - Resistant
I	I – Susceptible increasing exposure
S	S - Susceptible
